# Standard medical ethnobotany of Kohistan, North Pakistan

**DOI:** 10.1186/s13002-024-00704-w

**Published:** 2024-07-08

**Authors:** Muhammad Amin, Muhammad Abdul Aziz, Ajmal Khan Manduzai, Andrea Pieroni, Jawaher Alkahtani, Mohamed Ragab AbdelGawwad, Yusufjon Gafforov, Abdul Nazeer, Arshad Mehmood Abbasi

**Affiliations:** 1 Department of Environmental Sciences, COMSATS University Islamabad, Abbottabad Campus, Abbottabad, 22060 Pakistan; 2 Department of Environmental Sciences, Informatics and Statistics, Ca’ Foscari University of Venice, Via Torino 155, 30172 Venice, Italy; 3 University of Gastronomic Sciences of Pollenzo, Piazza V. Emanuele II, 12042 Bra/Pollenzo, Italy; 4 Department of Medical Analysis, Tishk International University, Erbil, Kurdistan 4401 Iraq; 5 Department of Botany and Microbiology, College of Science, King Saud University, P.O. 2455, 11451 Riyadh, Saudi Arabia; 6 Genetics and Bioengineering, Faculty of Engineering and Natural Sciences, International University of Sarajevo, 71210 Sarajevo, Bosnia and Herzegovina; 7 Central Asian Center for Development Studies, New Uzbekistan University, 100007 Tashkent, Uzbekistan; 8 Institute of Botany, Academy of Sciences of Republic of Uzbekistan, 100125 Tashkent, Uzbekistan

**Keywords:** Wild medicinal plants, Cross-culture, Fidelity level, Venn diagram: linguistics, Kohistan

## Abstract

**Background:**

This study was exclusively focused on the documentation and cross-cultural evaluation of ethnomedicinal knowledge (EMK) within the diverse linguistic groups of Kohistan situated between the Himalayan and Hindukush Mountain ranges in the north Pakistan.

**Methods:**

Data were gathered during the field survey (May 2022 to July 2023) through group conversations, semi-structured interviews, and on-site observation. Venn diagrams were employed to illustrate the comparative assessment of EMK, and different ethnobotanical indices were utilized to examine the data.

**Results:**

A total of 96 wild medicinal plant species (MPs) belonging to 74 genera and 52 botanical families were documented. The most reported MPs belong to the family Polygonaceae (11 species), followed by Asteraceae (9 species) and Lamiaceae (8 species). The ethnomedicinal uses of *Leontopodium himalayanum*, *Pedicularis oederi*, *Plocama brevifolia*, *Polypodium sibiricum*, *Pteridium esculentum*, *Sambucus wightiana*, *Solanum cinereum*, *Teucrium royleanum*, *Rhodiola integrifolia*, *Aconitum chasmanthum* were reported for the first time in this region. Among the reported taxa herbaceous species were dominated (72%), followed by trees and shrubs (17% and 10%, respectively). Digestive problems (40 taxa and 114 use reports) and skin disorders (19 taxa and 549 use reports) were the most cited disease categories, whereas *M. communis, M. longifolia, Ajuga integrifolia, Ziziphus jujuba,* and *Clematis grata* exhibited the highest percentage fidelity levels. Out of 109 documented medicinal uses, a mere 12 were shared across all linguistic groups, and Bateri emerges as a notable outlier with the highest number of medicinal uses. In addition, a significant homogeneity was noted in the reported botanical taxa (61 species) among different linguistic groups. However, since the last decade biocultural heritage of Kohistan is facing multifaceted risks that need urgent attention.

**Conclusion:**

Our findings could be valuable addition to the existing stock of ethnomedicinal knowledge and may provide ethnopharmacological basis to novel drug discovery for preexisting and emerging diseases prioritizing detailed phytochemical profiling and the evaluation of bioactive potential.

## Introduction

Kohistan region is an important hotspot of biocultural diversity and could be thus declared a biocultural refugium. The region has been a crossroad for the exchange of biocultural knowledge between South, Southwestern Asia, and Central Asia. The close sociocultural interactions among the different ethnic communities within the region and then the invasion of modernization and infrastructural developments pose a clear challenge to local ecological knowledge within each community. In the face of sociocultural as well as environmental change, the local ethnobotanies are changing and in some case the cultural dominancy has driven a change to make the local knowledge blended with exotic knowledge and this phenomenon is well understood in ethnobiology. The multiculturality and multilingualism of the region make the area a viable place to study human–plant interactions. It is worth mentioning that the remoteness of the area has stood out a reason for scientific research in the study area that may have potentially allowed the local communities to retain cultural practices alive and distinct around the local flora.

Our research on food ethnobotany recorded substantial body of local plant knowledge among the different ethnolinguistic groups living across the Kohistan region [1]. The current ethnobotanical survey has primarily been guided by our food ethnobotanical research work in the region. Our research has indicated significant level of idiosyncrasy or heterogeneity in local knowledge on food plants among the studied communities and considering these specific patterns of knowledge retention or mobilization within the different groups. We have hypothesized that some important debates especially related to human ecology could be constructed and could come with more concrete results on the pattern of local knowledge mobilization which we might have missed to derive from the results of our previous food ethnobotanical studies. Additionally, it may also give rise to new and more stimulating debate related to context-based social anthropology while discussing the local knowledge systems on medicinal plants within these specific communities.

North Pakistan has been extensively researched in ethnobotanical literature; however, the Kohistan region is still underexplored in the existing literature. The literature indicates specific trends in sharing local plant knowledge among the various ethnic groups. We believe that the medical ethnobotany and its related dynamics are a different subject area then food ethnobotany, and therefore, studies on the medical ethnobotany could provide new insights to enrich the anthropological discussion around the use of local medicinal plants. Moreover, culturally guided uses of plants are an important subject in medical anthropology and therefore the intertwining of medical anthropology and medical ethnobotany plays a pivotal role in understanding the dynamics how the local knowledge systems persist and evolve within modern societies.

In Kohistan, the traditional ecological knowledge including the plant knowledge is highly challenged by the new developing infrastructure that impact the local subsistence and ecological practices, and this in turn has negatively impacted the local knowledge systems. For instance, the ongoing mega projects such as Dassu and Basha Hydropower Projects and several small projects in different valleys of Kohistan are affecting the lifestyle of the young generation significantly. Slowly but surely, allopathic medicines are also integrating with traditional healthcare in this region. Specifically, recent developments under the China–Pakistan Economic Corridor (CPEC) project are one of the key factors diverting the attention of the young generation, thus recording the ethnomedicinal knowledge in Kohistan essential. Specifically, documenting the medical ethnobotany of the region will have a crucial role in understanding how different cultures use plants, and how these uses are guided by social, environmental, and economic factors in shaping human–plant relationship. The aim of the current study is that the medical ethnobotany of the ethnolinguistic groups in Kohistan would provide a holistic view; how human interact with their natural environment? The study will preserve new knowledge on scientific background and will give new directions to the already existing literature as we have seen that many Pakistani ethnobotanical studies have been conducted but lacking the best and standard practices to reach the minimum level of scientific integrity. Our study undertakes clear methodological approach—has been conducted with specific objective to address the proposed research questions. We have documented and compared the ethnomedicinal knowledge on wild medicinal plant species among different linguistic groups of Kohistan region in the north Pakistan.

The specific objectives of the study were:

1. Documentation of the local names and medicinal use of wild plants!
2. Cross-cultural analysis and comparison of the quoted plants used in traditional health care system among the different ethnic groups.

## Methodology

### Description of the study area

**Fig. 1:**
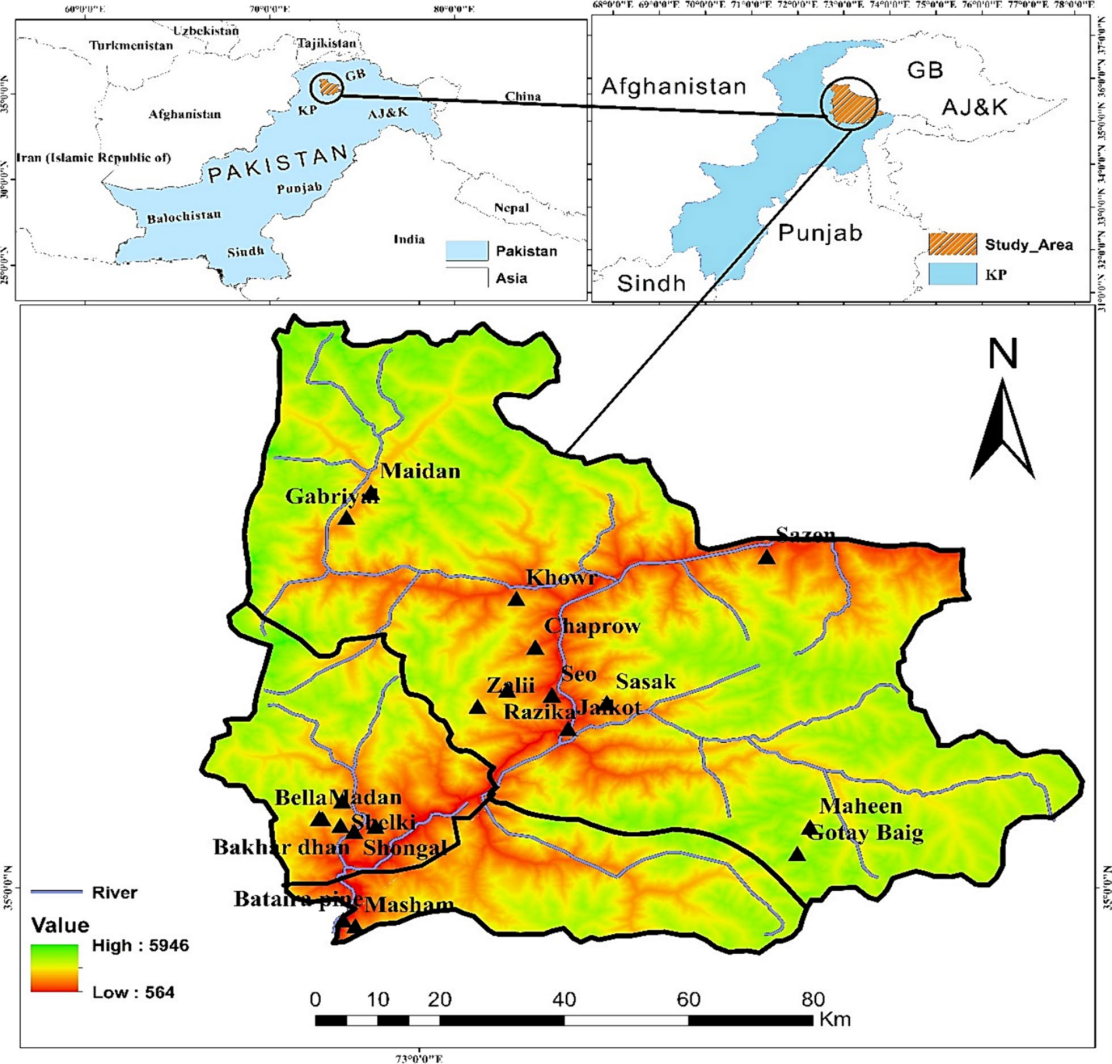
Map illustrating study area and survey sites

Kohistan region lies between 35° 16′ 16′′ N latitude and 73° 13′ 24′′ E longitude in the sub-humid eastern and wet mountain zones of north Pakistan [2]. Kohistan is subdivided into three districts, namely Kohistan Upper, Lower Kohistan, and Kolai Pallas Kohistan, as dissected by the Indus River (Fig. 1). Characterized by their geographical location, these districts are richly endowed with natural beauty, including majestic mountain vistas, fertile valleys, and vast expanses of biodiversity. Kohistan is distinguished by a dry and cold climate in the winter and a hot and dry climate in the summer. In the valleys, rainfall is prevalent during the winter, while snowfall is observed in alpine pastures and hilly areas [3].

The vegetation of Kohistan falls into dry and moist temperate forests areas [4, 5]. The primary objective of the present study was to conduct a comprehensive investigation on medicinal uses of wild plant species among different linguistic groups, including Kohistani, Shinaki, Bateri, Pashtoon, and Gujjars, residing in three districts of Kohistan. These linguistic groups, despite their unique dialects and varying lifestyles, do share certain commonalities in religious belief systems and dialect similarities, thus contributing to the diverse cultural mosaic of Kohistan. Additionally, we have discussed in detail the history and ethnolinguistic diversity of Kohistan in our previous article [1]. Agriculture, livestock rearing, and weaving constitute the primary economic activities of the inhabitants of Kohistan, significantly contributing to the region’s economic vitality [2]. Kohistan is the most remote region of Pakistan and lacks modern health facilities. Even in the contemporary era, this region lacks hospitals, necessitating inhabitants to travel over 150 km and endure a journey of at least seven hours to reach the nearest health facility in Abbottabad. Consequently, the inhabitants of this region rely mainly on plants and animals in their environment for sustenance and as components of their indigenous healing practices [6].

### Valleys and linguistic groups in Kohistan

**Fig. 2:**
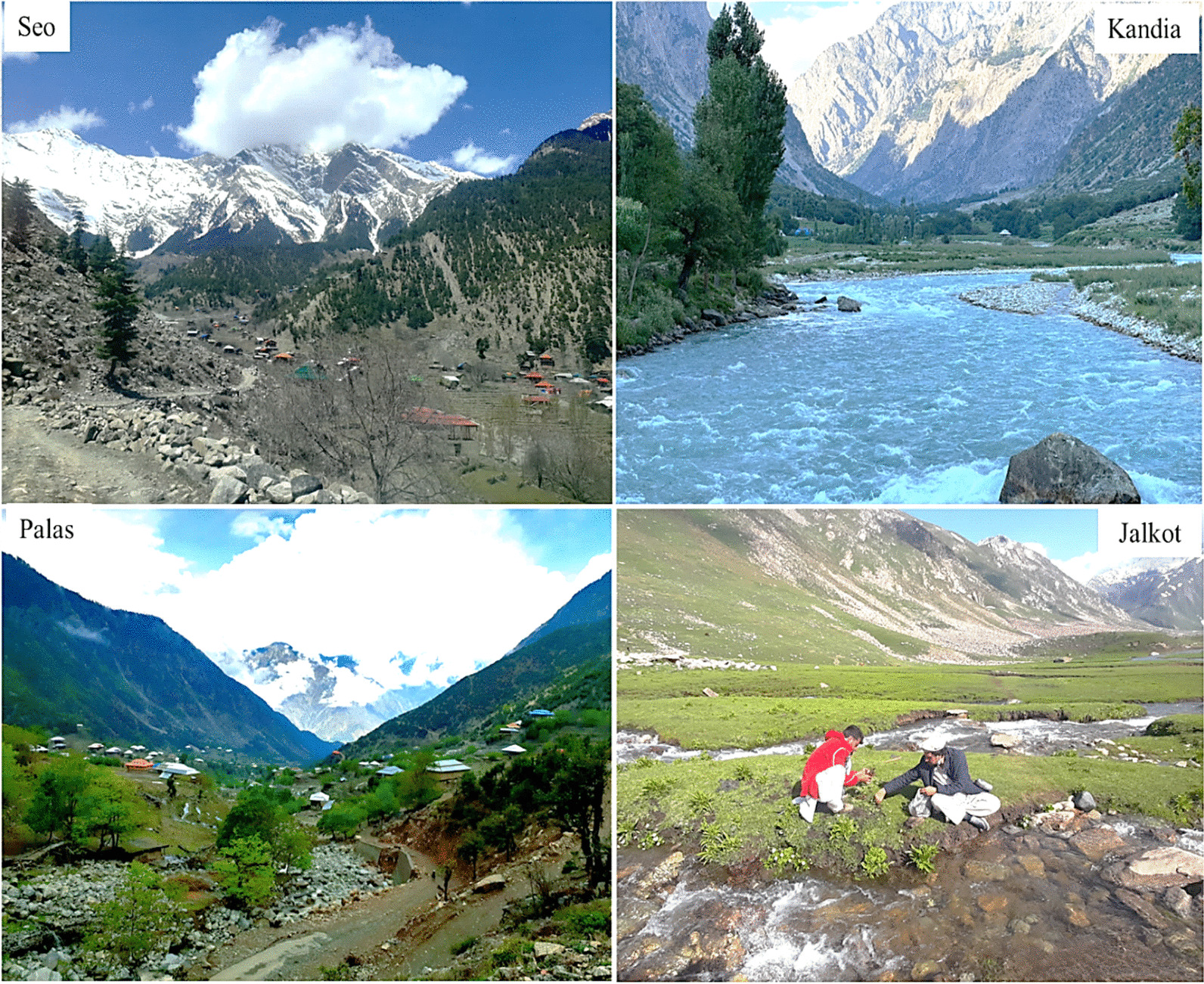
Panoramic view of different valleys in Kohistan

The Indus River splits Kohistan into eastern and western parts. Seo, Kandia, and Dubair are three major valleys on the western side, while Jalkot, Palas, and Kolai valleys lie on the eastern side (Fig. 2). In western part of Kohistan, local communities which are known as “Kohiste” speak Kohistani language in different dialects like Shuthun, Maiya, Indus Kohistani, and Abasin Kohistani. The history of Kohistani people and their language is controversial, as different people assume diverse ancestors of Kohistani. It has been documented that Kohistani language belongs to the Dardic group of Indo-Aryan languages [7–11]. According to Rensch [7], Kohistani language linking with the Torwali language is spoken in Swat valley. However, there is lack of further details on the origin of Indus Kohistani language.

Local inhabitants in Jalkot, Palas, and Kolai on the eastern side of Kohistan are known as “Shinaki” and they speak Shina language. Shina belongs to the Dardic language family which is a subgroup of the Indo-Aryan linguatuliasis. Phylogenetically, Shina has strong associations with Indo-Aryan, Dravidian, Austro Asiatic, and Tibeto Burman and is written in the “Persio-Arabic script.” Some researchers believe that Shina is Brahmi script which was originally used in the era of Ashok in 3rd BC [12]. Shina language has different dialects which are spoken in different valleys of Kohistan. It is the most spoken language in different valleys of Gilgit-Baltistan, Jammu and Kashmir, Ladakh regions of Pakistan, India, and China [9, 13, 14]. The different dialects of Shina language, for instance, Kohistani, Astori, Chilasi, Gilgiti, Hunzai, and Diameri, are mainly based on local influence, geography, and topography of valleys [13–15].

Bateri, Pushto, and Gujjari linguistic groups are minorities in Kohistan and live in specific areas. Bateri community live in Batera Pain and Batera Bala villages on the east bank of the Indus River in Kolai Pallas Kohistan. Bateri language also known as Bateri Kohistani and Batarwal language belongs to the Dardic language branch of Indo-European family. Beside Kohistan, Bateri is also spoken in Jammu and Kashmir region of India [7]. Pushto language belongs to the eastern Iranian language, a subgroup of Aryan linguistics family, and is further divided into Iranian and Indian branches. In Pakistan, the Pushto speaker has different accents and dialects [7, 16, 17]. Although Pushto linguistic group is limited in the Indus Kohistan region, Pashtuns (Pushto speakers) spread almost in all parts of Pakistan, and specifically in the north province “Khyber Pakhtunkhwa.” In Indus Kohistan, Pashtuns are common at Shongal and Jag villages in Dubair valley. The nomadic Gujjars live in the most remote area of each valley on both sides of Kohistan. It has been reported that Gujjars came to this region from Mongolia at the time of Mongol incursion on India in the fifth century [10]. These tribes speak Gujjari, which accounts for 10.5% compared to other Kohistani and Shina languages [7, 8].

### Field survey

**Fig. 3:**
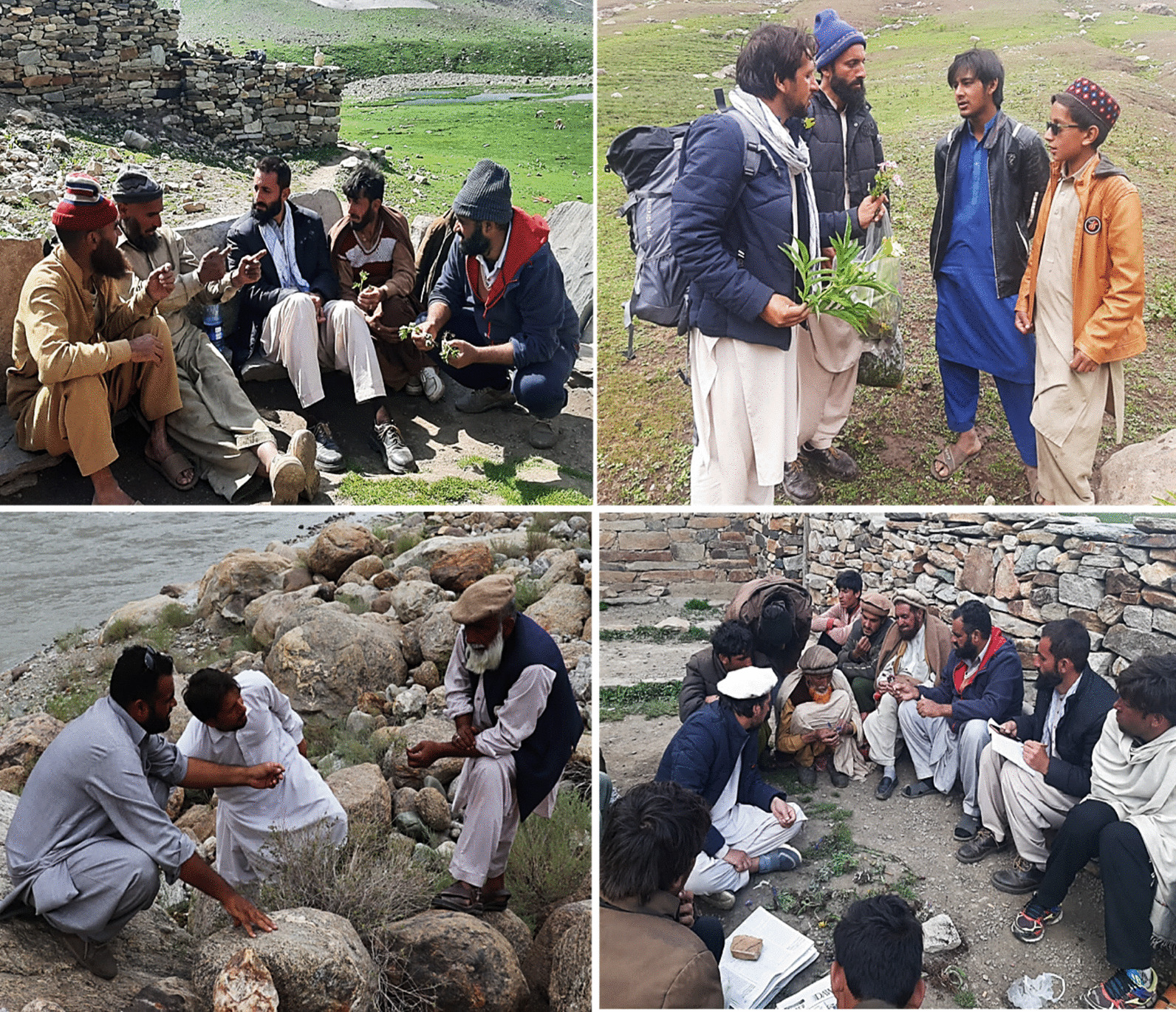
Authors collecting information during filed survey in different areas of Kohistan

Twenty study sites/villages were selected to conduct field survey from May 2022 to July 2023. Study sites’ selection was based on their ethnicity, linguistic groups, vegetation or forest types, and elevation zones. While conducting the field survey, we meticulously observed the ethical guidelines as recommended by “International Society of Ethnobiology” [18]. These guidelines emphasize the importance of maintaining respect for different cultures, acknowledging local knowledge, and preserving biodiversity. Verbal consent of the participants as reported previously [19, 20] was taken for data collection, photography, and sharing their knowledge and picture with the public. As demonstrated earlier [21], informants between 20 and 80 years of age were selected using the snowball approach. The study participants chosen for the survey have a long-lasting relationship with the natural environment and the local flora and were recognized as experienced in local medicinal plant knowledge. Male field researchers co-authoring this article recruited male study participants in the interviews (Fig. 3) since female informants could not be approached due to cultural/religious issues; therefore, the first author interviewed only those women who were his relatives. Moreover, it is also important to note that our respondents were not traditional healers or doctors but laypeople, i.e., experts in local plant knowledge who gained it orally from their elders. In the particular social and cultural context of the study area, women participants could be taken for the interviews if they local communities are guided better and explain the purpose of the study with the help of a local guide.

From each linguistic group, 15–20 informants including male and female were selected based on their age, gender, linguistic group, experience, and knowledge (Table 1). In Kohistan, open communication between male and female is strictly restricted, except close family members. With the help of female members or relatives from his family, the first author collected information from female respondents who reside in Kohistan. Data were collected by using field observation, semi-structured interviews, and group discussions as explained earlier [22]. The interviews as well as the group discussions were conducted in the local languages (Kohistani, Shina, Bateri, Pushto, and Gujjari). To ensure comprehension, native speakers of these languages and interpreters were engaged. The gathered information was subsequently translated into English, thus facilitating comparative analysis and interpretation.

**Table 1:** Characteristics of the targeted localities and study participants

Sites	Forest types	Ethnic group	AH	SA	EnR./ExR	Linguistic group	Villages	El. (m,a,s,l.)	NH	NI (M/F)	Age
Seo valley	Himalayan dry and moist temperate forests	Seowos (96%)	Sixteenth–seventeenth century	Pastoralism, animal husbandry and farming	Endogamic, rarely exogamic	Kohistani	Seo	878	320	13/5	15–60 years
							Razika	1830	275	15/3	
							Chaprow	1316	35	10/4	
		Gujjars/Guzar (4%)	NR	Pastoralism and animal husbandry	Exogamic	Gujjari	Zalii	3093	37	12/3	
Jalkot valley	Himalayan dry and moist temperate forest, and alpine pastures	Shinaki/Jalkoti (90%)	Sixteenth–seventeenth century	Pastoralism, animal husbandry, farming, and mining	Endogamic, rarely exogamic	Shina	Maheen	3503	25	15/0	20–80 years
							Sasak	2001	120	13/1	
							Sazen	1415	154	14/0	
							Jalkot	892	250	15/2	
		Gujjars/Guzar (10%)	NR	Pastoralism and animal husbandry	Exogamic	Gujjari	Gotay Baig	4741	25	9/0	
Kandia valley	Himalayan dry and moist temperate forests	Kheloos (85%)	Sixteenth–seventeenth century	Pastoralism, animal husbandry and farming	Endogamic, rarely exogamic	Kohistani	Khowr	1633	75	11/1	20–80 years
							Gabriyal	2113	286	12/2	
		Gujar/Guzar (15%)	NR	Pastoralism and animal husbandry	Exogamic	Gujjari	Maidan	2655	85	11/0	
Kolai valley	Himalayan dry and moist temperate forest, and alpine pastures	Kulooj (95%)	Sixteenth–seventeenth century	Pastoralism, animal husbandry and farming	Exogamic	Shina	Bella	2232	45	10/3	20–70 years
							Madan	2226	250	14/1	
		Bahtooj (5%)	NR	Pastoralism, animal husbandry and farming	Endogamic, rarely exogamic	Bateri	Bateri pine	625	230	13/4	
							Masham	1810	170	9/3	
Dubair valley	Himalayan dry and moist temperate forest, and alpine pastures	Shongali (5%)	NR	Pastoralism, animal husbandry and farming	Exogamic	Pushto	Shongal	1612	120	12/2	20–60 years
		Jaagi (5%)	NR				Jaag Kali	1548	160	11/3	
		Dubairi (90%)	Sixteenth–seventeenth century	Pastoralism, animal husbandry and farming	Endogamic	Kohistani	Bakhar dhan	2286	95	13/1	
							Shelki	2036	65	9/2	

*AH* arrival history in the area, *SA* subsistence activities, *EnR* endogamic rules, *ExR* exogamic rules, *El* elevation, *NH* number of households, *NI* number of informants, *M* male, *F* female, *AG* age groups, *NR* no record

We adopted a mixed approach for selecting the informants. The survey started with participants selected through random sampling, and then, once we became familiar with the study area, we adopted the snowball technique. The duration of the interviews varied, i.e., in some cases, it ended after 20 min, while in others, it lasted for hours. The participants with long experience of nature and who remained in the study area for decades were preferred. Interviews were conducted in public gathering places, local shops, and fields, mainly after prayer near mosques where the local population used to gather and interact. Some people were also interviewed while working in the fields. The information collected from the interviewees focused on the local names of medicinal taxa, parts used, diseases treated, and modes of preparation and application. Free listing was used to obtain a thorough knowledge of the therapeutic uses of the quoted plants. Initial free listing was attempted, but it was usually short and rarely succeeded.

### Plant specimen collection and processing

Medicinal plant species enlisted in Table 2, used by the inhabitants of the study area, were collected during the field survey. The plant specimen were identified by expert taxonomists and with the help of Flora of Pakistan [23]. Botanical nomenclature and family name of the identified specimen were further confirmed with the help of international data base of plant species “The World Flora Online” https://www.worldfloraonline.org/. All specimens were properly dried, poisoned, and mounted on herbarium sheets, and the voucher specimens were deposited in the herbarium at COMSATS University Islamabad, Abbottabad Campus, for future records.

**Table 2:** Ethnomedicinal application of wild plants of Kohistan

**S. #**	Scientific name/ Family/Local name/ voucher number	Habit	Part(s) use	Preparation	Application	Diseases treated	Disease categories	FC	RFC	PRMUP	PRMUN
1	*Ajuga integrifolia* Buch. -Ham Lamiaceae Bhuti^K,S,G^ CUHA-06	Herb	Lvs./Stm	Powder, Decoction	Oral	Fever, skin infection, to purify blood	General and unspecified, Skin, Cardiovascular	14	0.05	24	24
2	*Aconitum chasmanthum* Stapf ex Holmes Ranunculaceae Sakhat Moril^S^ CUHA-405	Herb	Rt	Powder mixed in maize flour	Oral	Anti-rodents	Others	15	0.05		25
3	*Artemisia gmelinii* Weber ex Stechm Asteraceae Tanda Tarkha^P^ CUHA-237	Herb	Lvs	Paste and fresh leaves	Topical	Wounds healing, injuries	Skin	14	0.05	26	
4	*Artemisia stechmanniana* Besser Asteraceae Dada Tarkha^P^ CUHA-239	Herb	Lvs,	Tea	Oral	Fever, flue, cough, corona symptoms	General and Unspecified, Respiratory	14	0.05		27
5	*Astragalus anisacanthus* Boiss Leguminosae Muskanda^K^, Chokanda^G^ CUHA-13	Herb	Rt	Toothbrush, Paste	Topical	To clean teeth and mouth gums	Digestive	18	0.06	28	
6	*Berberis lycium* Royle Berberidaceae Shoongloo^K,S^, Shuglo^B^, Koaray^P^ CUHA-204	Shrub	Rt	Powder with egg white	Topical and Oral	Wounds healing and Backache	Skin, Musculoskeletal	12	0.04	29	
7	*Bergenia stracheyi *(Hook.f. & Thomson) Saxifragaceae Kala par^K^, Kurat^S^, Rechowoo^G^ CUHA-187	Herb	Rt	Powder	Oral	Chest infection, stomach disorder, and backache	Respiratory, Digestive, Musculoskeletal	9	0.03	30	
8	*Bergenia ciliata* (Haw.) Sternb Saxifragaceae Kala par^K^, Kurat^S^, Rechowoo^G^ CUHA-14	Herb	Rt	Powder	Oral	Chest infection, stomach disorder, and backache	Respiratory, Digestive, Musculoskeletal	15	0.05	29	
9	*Buxus wallichiana* Baill Buxaceae Neek^B^ CUHA-250	Shrub	Lvs	Paste	Oral	Constipation	Digestive	12	0.04	31	
10	*Caltha palustris* L Ranunculaceae Patara^G^ CUHA-251	Herb	Lvs	Powder with butter	Oral	Intestinal wounds	Digestive	9	0.03	32	
11	*Cannabis sativa* L Cannabaceae Bhang ^S,G^, Fat kanda^B^ CUHA-16	Herb	Lvs	Paste with maize flour	Topical	Body weakness and indigestion	Digestive	15	0.05	33	
12	*Cedrus deodara* (Roxb. ex D.Don) Pinaceae Beech^K^, Plolz^S^, Low ^G^ CUHA-17	Tree	Rt./Stm./Rn	Oil	Topical	Skin infection, against pests/insects, as anti-hair fall, mouth gums, stomach disorders	Skin, Digestive, Others	14	0.05	30	
13	*Celtis caucasica* Willd Cannabaceae Meyoon^K,S^, Dodoo^B^ CUHA-401	Tree	Fr	Powder	Oral	Allergy	Others	14	0.05	31	
14	*Chrysojasminum humile* (L.) Banfi Oleaceae Tobkio^S^, Naltah ^B^ CUHA-277	Herb	Rt	Powder	Oral	Ringworms	Digestive	15	0.05	36	
15	*Cirsium arvense* (L.) Scop Asteraceae Horal^G^ CUHA-27	Herb	Ap./Rt	Decoction and Powder	Oral	Typhoid fever, and to heal wounds	General and Unspecified, Skin	15	0.05	37	
16	*Cirsium verutum* Spreng Asteraceae Zach^K^, Jaacha^S^, Zheheea^B^, Kenare^G^ CUHA-254	Herb	Lvs	Fresh Part/Powder	Oral	Fever	General and Unspecified	11	0.04		38
17	*Clematis grata* Wall Ranunculaceae Zulto^K^ CUHA-26	Climber	Lvs	Powder	Oral	Urinary disorder and bladder infection	Urological	12	0.04	32	
18	*Clinopodium vulgare* L Lamiaceae Shakbar buti^K^ CUHA-255	Herb	Lvs	Powder	Oral	Internal wounds	Digestive	14	0.05	39	
19	*Daphne mucronata* Royle Thymelaeaceae Laatar ^B^ CUHA-259	Shrub	Fr	Paste	Topical	Face stains	Digestive, Skin	12	0.04	40	
20	*Datisca cannabina* L Datiscaceae Karayee^K^, Kalbeer^S^, Barsatra^B^, Kurkoron^G^ CUHA-30	Herb	Rt	Paste	Topical	Joint swelling	Musculoskeletal	14	0.05	41	
21	*Dodonaea viscosa* Jacq Sapindaceae Shonth^K^, Ashad^B^, Goodni^G^ CUHA-33	Shrub	Lvs	Extract	Orally	Throat infection	Respiratory	13	0.05	42	
22	*Duchesnea indica* (Andrews) Teschem Rosaceae Bhangros^K^, Magros^S^, Muabros^B^, Dharti Mian^G^, Bhangrus^P^ CUHA-263	Herb	Fr	Fresh Part	Oral	Indigestion	Digestive	12	0.04	43	
23	*Dysphania botrys* (L.) Mosyakin & Clemants Amaranthaceae Jamama^B^ CUHA-253	Herb	Lvs	Powder	Oral	Abdominal warm	Digestive	17	0.06	32	
24	*Erigeron canadensis* L Asteraceae Lindi^K^, Kasar^S^ CUHA-28	Herb	Ap	Paste	Topical	Toothache	Digestive	14	0.05	42	
25	*Ficus palmata* Forssk Moraceae Phah^K,B^, Pagoye^S^, Camera^G^ CUHA-37	Tree	Lvs./Milk	Paste	Topical	spots or stain on face, to heal wounds	Skin	12	0.04	34	
26	*Geranium himalayense* Klotzsch Geraniaceae Rathan Jok^G,S^ CUHA-267	Herb	Ap	Powder	Oral	stomach pain	Digestive	11	0.04	45	
27	*Hypericum oblongifolium* Choisy Hypericaceae Khan kawa^B^ CUHA-271	Herb	Lvs	Powder	Oral	Backache	Musculoskeletal	12	0.04	45	
28	*Hypericum perforatum* L Hypericaceae Chai thigo^K^, Esperkaye^G^ CUHA-41	Herb	Lvs	Decoction and Powder	Oral	Backache	Musculoskeletal	11	0.04	46	
29	*Indigofera tinctoria* L Fabaceae Kayth ^K^, Kasti^S^, Khati ^B^, Kheenthey^G^ CUHA-43	Shrub	Stm./Lvs	Toothbrush, Decoction	Oral	To strengthen teeth, paralysis	Digestive, Neurological,	12	0.04	47	
30	*Juglans regia* L Juglandaceae Choo^K^, Achoye^S^, Akhory^G^ CUHA-45	Tree	Brk	Fresh	Topical	To clean teeth, mouth gums	Digestive	14	0.05	48	
31	*Juniperus excelsa* M.Bieb Cupressaceae Cheli^S^ CUHA-279	Tree	Fr	Powder	Oral	Kidney inflation	Urological	15	0.05	49	
32	*Justicia adhatoda* L Acanthaceae Bhekar^S^, Bashee^B^ CUHA-190	Shrub	Lvs	Paste	Topical	Wounds healing	Skin	13	0.05	50	
33	*Leontopodium himalayanum* DC. Compositae Taliban kaleel^S^ CUHA-280	Herb	Lvs	Fresh Part	Oral	Abdominal pain	Digestive	15	0.05		
34	*Lepidium sativum* L Brassicaceae Tumera^S^,, Gulpecha^B^ CUHA-46	Herb	Ap	Decoction	Oral	Asthma	Respiratory	15	0.05	42	
35	*Mallotus philippensis* (Lam.) Müll.Arg Euphorbiaceae Kamla^S^, Kambila^B^ CUHA-284	Tree	Fr	Powder	Oral	Abdominal warms	Digestive	17	0.06	34	
36	*Melia azedarach* L Meliaceae Kamsal lagha^K^, Lagand^S^, Tora bekar^P^ CUHA-47	Tree	Lvs	Powder	Oral	Allergy	General and Unspecified	14	0.05	41	
37	*Mentha arvensis* L Lamiaceae Phepeel^S^, Podina^G^ CUHA-287	Herb	Ap	Powder	Oral	Diarrhea	Digestive	15	0.05	51	
38	*Mentha longifolia* (L.) L Lamiaceae Phemil^K^, Phepeel^S^, Zoon^B^, Podina^G^, velini^P^ CUHA-48	Herb	Lvs	Powder and Tea	Oral	Diarrhea and indigestion	Digestive	14	0.05	40	
39	*Mentha spicata* L Lamiaceae Phemil^K^, Podina^G^ CUHA-288	Herb	Lvs	Decoction	Oral	Diarrhea and indigestion	Digestive	14	0.05	40	
40	*Myrtus communis* L Myrtaceae Ambo^K^, Aob^S^ CUHA-26	Herb	Fr./Lvs	Powder	Topical	Eczema	Skin	18	0.06	48	
41	*Nepeta podostachys* Benth Lamiaceae Jurpe^K^, Pushmily^P^ CUHA-51	Herb	Lvs./Stm	Paste	Topical	Skin infections	Skin	15	0.05	52	
42	*Nerium oleander* L Apocynaceae Phool^K,B^ CUHA-292	Shrub	Lvs	Decoction	Topical	To kill pests and fungus	Others	17	0.06	31	
43	*Oreoseris gossypina* (Royle) X.D.Xu & V.A.Funk) Asteraceae Khoon^P^ CUHA-402	Herb	Rt	Powder	Topical	Intestinal wounds	Digestive			53	
44	*Oxalis corniculata* L Oxalidaceae Cheko daro^K^, Chiki rang^S^ CUHA-53	Herb	Lvs	Extract	Oral	Jaundice and hepatitis	Digestive	14	0.05	31	
45	*Oxyria digyna* Hill Polygonaceae Huli^S^ CUHA-294	Herb	Ap	Fresh Part/Powder	Oral	Constipation	Digestive	15	0.05	54	
46	*Pedicularis oederi* Vahl Orobanchaceae Khana Phor^S^ CUHA-296	Herb	Lvs	Powder	Oral	Indigestion and weakness	Digestive	15	0.05		
47	*Persicaria amplexicaulis* (D.Don) Ronse Decr Polygonaceae Roy^K^ CUHA-57	Herb	Lvs	Powder	Oral	Backache	Others	14	0.05	55	
48	*Persicaria capitata* (Buch. -Ham. ex D.Don) H.Gross Polygonaceae Maran Kash^K^ CUHA-58	Herb	Ap	Powder	Oral	Urinary tract infection and urinary bladder inflammation	Urological	18	0.06	56	
49	*Pistacia khinjuk* Stocks Anacardiaceae Khakow^K^, Kakoh^S^ CUHA-63	Tree	Lvs	Powder	Oral	Asthma and cough	Respiratory	14	0.05	57	
50	*Plantago lanceolata* L Plantaginaceae Cheelor^K,S,G^, Jabay^B^ CUHA-64	Herb	Lvs	Paste	Topical	Skin infections	Skin	15	0.05	58	
51	*Plantago major* L Plantaginaceae Cheelor^K,G^ CUHA-65	Herb	Lvs	Decoction	Oral	Skin infection	Skin	12	0.04	58	
52	*Plocama brevifolia* subsp. Brevifolia Rubiaceae Zoonr^S^, Jhoor^B^ CUHA-300	Herb	Ap	Paste	Topical	Blood clots and injuries	Blood	15	0.05		
53	*Podophyllum hexandrum* Royle Berberidaceae Shargoye^K^, Shingoy^S^, Khakori^G^ CUHA-66	Herb	Rt	Paste	Oral	Warts and other skin infections	Skin	14	0.05	59	
54	*Polygonatum multiflorum* (L.) All. Asparagaceae Aschawagi^K^ CUHA-67	Herb	Rt	Powder	Oral	Abdominal pain, dysentery	Digestive	14	0.05	60	
55	*Polygonum aviculare* L Polygonaceae Zat Gah^K^ CUHA-68	Herb	Rt	Powder	Topical	External wounds	Skin	18	0.06	61	
56	*Polypodium sibiricum* Sipliv Polypodiaceae Shaal^K^ CUHA-70	Herb	Ap./Lvs	Decoction/Powder	Oral/ Topical	Intestinal ulcers, to heal wounds or injuries, stomach disorder	Digestive, Skin	18	0.06		
57	*Primula elliptica* Royle Primulaceae Kamzoor Moril^S^ CUHA-302	Herb	Lvs	Ash	Topical	Mouth gums, diarrhea/ cough	Digestive, Others	15	0.05	62	
58	*Primula macrophylla* D. Don Primulaceae Moril^S^ CUHA-303	Herb	Lvs	Ash	Topical	Mouth gums	Others	15	0.05	62	
59	*Prunus persica* (L.) Batsch Rosaceae Aro^K,G^, Aar^S^ CUHA-74	Tree	Lvs	Infusion	Topical	Fungal and bacterial infections	Skin	17	0.06	54	
60	*Pteridium aquilinum* L. Kuhn Dennstaedtiaceae Asbo mut ^K^ CUHA-75	Herb	Lvs	Powder	Oral	Chest pain	Respiratory	14	0.05	63	
61	*Pteridium esculentum* (G.Forst.) Cockayne Dennstaedtiaceae Kongii^G^ CUHA-76	Herb	Ap	Powder	Oral	External wounds, tumors, internal injuries	Skin, Cancer, Digestive, Blood	12	0.04		
62	*Punica granatum* L Lythraceae Dango^K^, Daroye^S^, Deengo^B^, Daroo^G^, Anogora^P^ CUHA-78	Tree	Rnd./Rt	Decoction/Powder	Oral	External wounds, tumors, internal injuries, intestinal worms	Skin, Digestive, Blood	18	0.06	64	
63	*Quercus semecarpifolia* Sm Fagaceae Ban^K^, Bani^S^, Tarra^B^ CUHA-81	Tree	Fr	Fresh Part	Oral	Stomach problem, to strengthen bones	Digestive, Musculoskeletal	18	0.06	64	
64	*Rheum australe* D. Don Polygonaceae Chutiyal^K,S^ CUHA-311	Herb	Rt	Powder	Oral	Constipation	Digestive,	9	0.03	65	
65	*Rheum webbianum* Royle Polygonaceae Chutiyal Kamzoor^S,K^ CUHA-312	Herb	Rt	Fresh Part/Powder	Oral	Constipation	Digestive	9	0.03	66	
66	*Rhodiola integrifolia* Raf Crassulaceae Pechil^S^ CUHA-313	Herb	Ap	Powder with maize flour	Oral	Vomiting and indigestion	Digestive	9	0.03		
67	*Ricinus communis* L Euphorbiaceae Jamal ghota^S^ CUHA-157	Tree	Lvs	Fresh leaves	Topical	External wounds	Skin	11	0.04	42	
68	*Rubus fruticosus* Lour Rosaceae Ancha^K^ CUHA-88	Shrub	Lvs	Decoction	Oral	Diarrhea, dysentery	Digestive	12	0.04	32	
69	*Rubus niveus var. micranthus* (D.Don) H.Hara Rosaceae Ancha^K^, Zekeeny^S^ CUHA-89	Shrub	Lvs	Powder	Oral	Cough and fever	Respiratory, General and Unspecified	15	0.05		67
70	*Rumex acetosa* L Polygonaceae Chuki^S^ CUHA-318	Herb	Rt./Lvs	Extract	Oral	Jaundice and hepatitis	Digestive	15	0.05	68	
71	*Rumex hastatus* D.Don Polygonaceae Chowko^S^, Huli^G^ CUHA-92	Herb	Ap	Extract	Oral	Skin rashes, jaundice, and hepatitis	Skin, Digestive	15	0.05	42	
72	*Rumex nepalensis* Spreng Polygonaceae Hububal^S^ CUHA-319	Herb	Ap	Infusion and paste	Oral	Corona infection, constipation	Respiratory, Digestive	14	0.05	42	
73	*Rumex abyssinicus* Jacq Polygonaceae Markash^K^ CUHA-90	Herb	Rt	Decoction	Oral	Jaundice and hepatitis	Digestive	14	0.05		69
74	*Rumex dentatus* L Polygonaceae Babal^K^, Hububal^S^, Hulow^G^ CUHA-91	Herb	Rt	Paste with maize flour	Oral	Constipation	Digestive	14	0.05	42	
75	*Salix tetrasperma* Roxb Salicaceae Beyo^K,S^, Batkorol^S^ CUHA-94	Tree	Lvs	Decoction	Oral	Fever, common cold	General and Unspecified	17	0.06		
76	*Sambucus wightiana* Wall. Ex. Wight & Arn Adoxaceae Katool^K^, Ganala^S^, Kooh^G^ CUHA-95	Herb	Stm	Fresh Part	Topical/ Smoke	Fever, sickness	General and Unspecified	18	0.06		
77	*Selaginella*. Sp Selaginellaceae CUHA-403	Herb	Ap	Fresh Part	Inhalation	Fever, sickness	General and Unspecified	17	0.06	70	
78	*Seriphidium maritimum* subsp. *Maritimum* Asteraceae Salal^K^, Jund^S^ CUHA-404	Herb	Ap	Fresh Part	Inhalation	Mosquito repellent	Others	14	0.05	71	
79	*Silene conoidea *L Caryophyllaceae Bandakay^B^ CUHA-324	Herb	Lvs	Fresh Part	Oral	Poultry diseases ailments	Others	12	0.04		72
80	*Skimmia anquetilia* N.P.Taylor & Airy Shaw Rutaceae Nameer^K,S^ CUHA-326	Herb	Lvs	Powder and paste	Oral	Smallpox, to kill worms, as an appetizer	General and Unspecified, Others	11	0.04		73
81	*Solanum nigrum* Acerbi ex Dunal Solanaceae Kuchmacho^K,G^, Kachmach^S^ CUHA-97	Herb	Lvs	Extract/Powder	Oral	Urinary bladder inflammation and infection	Urological	14	0.05	41	
82	*Solanum villosum* Mill Solanaceae Kuchmacho ^KG^,Kachmach^S^ CUHA-330	Herb	Lvs	Extract/Powder	Oral	Urinary bladder inflammation and infection	Urological	17	0.06	74	
83	*Solanum virginianum* L Solanaceae Kanr Sha^B^ CUHA-331	Herb	Fr	Paste	Topical	Dandruff	Skin	17	0.06	75	
84	*Solanum miniatum* Bernh. Ex. Wild Solanaceae Ker Ker ^K^ CUHA-96	Herb	Lvs	Fresh Part	Oral	Mouth gums and toothache	Digestive	14	0.05	76	
85	*Solanum cinereum* R.Br Solanaceae Zacha rango^K^, Kool Zacha^S^ CUHA-406	Herb	Sd	Oil	Topical	Dandruff	Skin	17	0.06		
86	*Sonchus arvensis* L Asteraceae Cheer Gandal^B^ CUHA-332	Herb	Lvs	Extract	Topical	Mol	Skin	12	0.04	32	
87	*Tagetes minuta* L Asteraceae Midi^K^, Doldooli^S^ CUHA-101	Herb	Fl	Extract	Topical	Mosquito repellent	Others	14	0.05	77	
88	*Taraxacum campylodes* G.E.Haglund Compositae Handh^S^ CUHA-336	Herb	Lvs	Powder	Oral	Liver disorder	Digestive	15	0.05	21	
89	*Taxus wallichiana* Zucc Taxaceae Thoon^K^, Baray^P^ CUHA-102	Tree	Brk	Powder	Oral	Cough	Respiratory	11	0.04	78	
90	*Teucrium royleanum* Wall Lamiaceae Plaki Thago^K^, Satqa Boti^P^ CUHA-338	Herb	Ap	Powder	Oral	Obesity	Digestive	11	0.04		
91	*Thymus linearis* Benth Lamiaceae Esperki ^K,P^, Kalay de Jaar^S^ CUHA-339	Herb	Lvs	Infusion	Oral	Abdominal pain and intestinal worms	Digestive	11	0.04	71	
92	*Trifolium pratense* L Leguminosae Kana Eshpet^K^, Shotal^S,G^ CUHA-103	Herb	Lvs	Powder	Oral	Cough, cold, and asthma	Respiratory	14	0.05	64	
93	*Valeriana jatamansi* Jones Caprifoliaceae Mushkebala^K,G^ CUHA-105	Herb	Ap	Powder	Oral	Fever and the common cold	General and Unspecified, respiratory	15	0.05	79	
94	*Verbascum thapsus* L Scrophulariaceae Khardak ^K,B^, Kuthay Kum^S^, Khotikam^G^ CUHA-106	Herb	Fl./Lvs	Powder	Oral	Cough, cold, and asthma, labor pain and wounds	Respiratory, Digestive	12	0.04	34	
95	*Zanthoxylum armatum* DC Rutaceae Tember^B^ CUHA-199	Shrub	Fr	Powder	Oral	Flue	Respiratory	12	0.04	80	
96	*Ziziphus jujuba* Mill Rhamnaceae Sagee^K^, Sazen^S^, Sageen^G^ CUHA-112	Tree	Sd	Powder	Oral	Blood pressure, to purify blood	Cardiovascular, Blood	13	0.05	81	

*K* kohistani, *S* shina, *B* bateri, *P* pushto, *G* gujjari, *PRTU* previously reported as ethnobotanical use, *PRMP* previously reported as a medicinal plant, *FC* frequency of citation, *RFC* relative frequency of citation, *UV* used value, *Ap* aerial part, *Lvs* leaves, *Rt* roots, *Stm* stem, *Fl* flower, *Fr* fruit, *Sd* seed, *Brk* bark, *Rn* resin, *Rnd* rind

### Data analysis

Filed data on ethnomedicinal uses of the reported botanical taxa were processed using MS Excel. Furthermore, different ethnobotanical indices, i.e., relative frequency citation (RFC), informant consent factor (ICF), and fidelity level (FL), were also computed. Data were presented in tabulated and graphical format with the help of Sigma Plot v12. Comparative assessment of data among different linguistic groups was presented as Venn diagrams drawn using free software available at http://bioinformatics.psb.ugent.be/webtools/Venn/[05 August 2023]. To find out novel uses of plant species, a comparison was made between the current ethnobotanical data and previously reported literature from Pakistan and neighboring countries [4, 21, 24–80], as given in Table 2.

### Relative frequency citation (RFC)

The measured value of RFC demonstrates the importance of each plant species among the local inhabitants of an area. RFC is calculated based on the frequency of citation (FC) of each species, which indicates the number of informants citing the use of plant species for a specific purpose. We used the following equation to calculate RFC value of the reported plant species as described earlier [81].

1 $${\text{RFC }} = \frac{{{\text{FC}}}}{N}\left( {0 < {\text{RFC}} < 1} \right)$$

FC indicates the frequency of citation of each species by respondents, and *N* is the total number of respondents participating in the survey.

### Informant consensus factor (ICF)

The informant consensus factor is used to analyze the degree of agreement of the respondents about each use category. ICF of the reported species was computed by using below mentioned formula as reported earlier [81, 82].

4 $${\text{ICF }} = \frac{{\left( {{\text{Nur}} - {\text{Nt}}} \right) }}{{\left( {{\text{Nur}} - 1} \right) }}$$

Nur is the number of use reports in each use category, and Nt is the number of plant species used for that category.

### Fidelity level (FL)

The fidelity level specifies ratio among the number of respondents who mentioned use of a plant for a particular purpose and the total number of informants who mentioned the use of the plants for any purpose regardless of any use category. The FL values ≈ 100% are obtained for plants having almost all use-mentions refer to same purpose, whereas low FL is obtained for the plant species that have multipurpose uses [83, 84]. The fidelity level of the reported species was calculated using the formula as given here:

3 $${\text{FL }}\left( \% \right) \, = \frac{{{\text{Ip}}}}{{{\text{Iu}}}} \times {1}00$$

Ip is the number of respondents who reported the use of a plant species for a specific purpose, and Iu is the total number of respondents who mentioned that plant species for any purpose.

## Results and discussion

### Features of the targeted localities and respondents

Data were meticulously gathered for the current ethnomedical study from 20 villages dispersed across five distinct valleys in the captivating Kohistan region: Seo, Jalkot, Kandia, Kolai, and Dubair (Table 1). The survey spanned an altitudinal range from 625 m above sea level (m.a.s.l.) in Bateri pine village to 4741 m.a.s.l. in Gotay Baig village. The vegetation cover in the surveyed area exhibited a spectrum from dry temperate to moist temperate, and subalpine to alpine pastures, mirroring the altitudinal variations.

Kohistan’s entire population adheres to Sunni Muslims, with historical records indicating their settlement in the region from the sixteenth to seventeenth century and conversion to Islam occurred during the eighteenth century. According to Fredrik Bart (1956), the Chinese Pilgrims traveled through the Indus Kohistan region. Although the Chinese pilgrim reported about the difficult trains of the routes, it did not mention about the inhabitants of Kohistani [10, 85]. Notably, the Kohistani people differentiate themselves in terms of ethnicity, language, and culture from neighboring populations in the Swat, Dir, Gilgit, and Baltistan regions of Pakistan. Their beliefs underscore distinctions between both Pashtun and other local communities in Gilgit-Baltistan. Most of the local population in Kohistan faces literacy challenges and is engaging in subsistence activities such as pastoralism, animal husbandry, farming, and mining.

In the present study, data were collected from 281 informants of 20–80 years of age. Ethnically, these informants were classified into distinct groups, including Seowos, Gujjars, Shinaki, Jalkoti, Kheloos, Kulooj, Bahtooj, Shongali, Jaagi, and Dubairi. These groups communicate primarily in the Kohistani, Shina, Gujjari, Bateri, and Pushto languages. Each linguistic group exhibits a unique culture and predominantly resides in different valleys, except for the Gujjars, who are dispersed across all valleys. Most of these linguistic or ethnic groups practice endogamy, with rare instances of exogamy, except for the Gujjars and Bateri, who engage in exogamous relationships with other ethnic groups. Unfortunately, the latter two groups often experience marginalization within the Kohistan region. This intricate social fabric reflects the diversity and historical evolution of the Kohistani people.

### Diversity of wild medicinal plant species

The wild medicinal plant species (WMPs) have played a significant role in the mountain communities’ healthcare practices for centuries and continue to do so today. The utilization of WMPs has been a longstanding tradition in the healthcare practices of the Kohistan region. This emphasizes the reliance of the mountain communities in this region on various plant species to meet their healthcare needs. Specifically, the WMPs play a crucial role in the local healthcare practices and are considered an integral part of the traditional medicine system of Kohistan.

According to the data presented in Table 2, the inhabitants of Kohistan use 96 WMPs, which belong to 74 distinct genera and 52 botanical families. Among the botanical families, Polygonaceae was the most prevalent, comprising 11 WMPs, followed closely by Asteraceae, Lamiaceae, Solanaceae, and Rosaceae, each contributing 10, 8, 5, and 4 WMPs, respectively. The extensive use of WMPs from the Polygonaceae family can be attributed to their diverse medicinal properties, such as anti-inflammatory, wound-healing, and antibacterial potential that make them effective in treating a wide range of ailments [86]. Interestingly, our findings align with previous studies conducted in the northern parts of Pakistan [22, 87]. These studies have also reported the dominance of Polygonaceae and Lamiaceae families in the Gilgit-Baltistan region, Kohistan, and surrounding areas is due to their widespread ecological distribution [60, 88, 89].

**Fig. 4:**
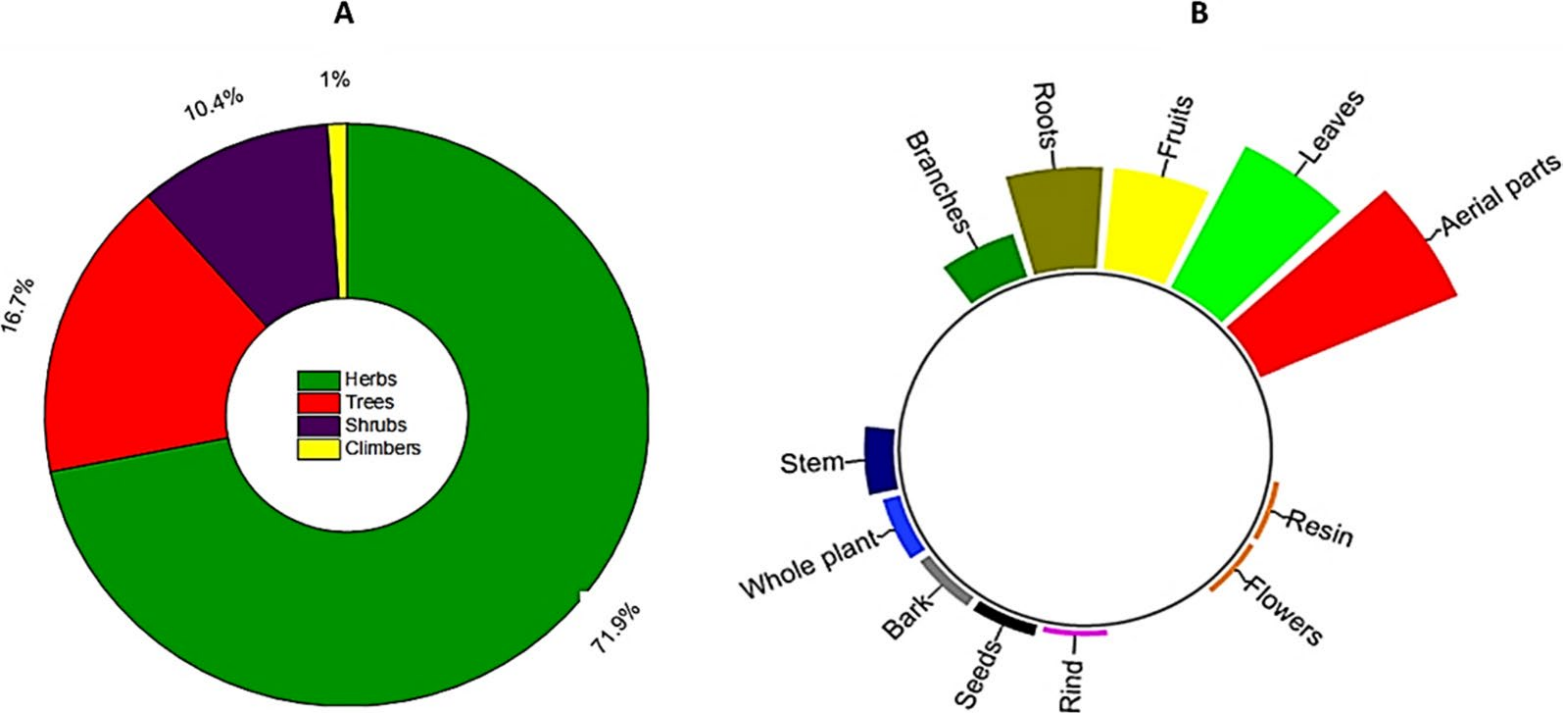
**A** Different life forms **B** part(s) used of the reported WMPs

As depicted in Fig. 4A, the reported WMPs consist most of herbs (71.8%), followed by trees (16.6%) and shrubs (10.4%). These findings align with the geographical features of the Kohistan region, which predominantly consists of expansive pasture lands adorned with a variety of lush green herbs. Additionally, the local inhabitants of Kohistan exhibit a preference for collecting and utilizing herbaceous WMPs from their surroundings due to their accessibility and numerous health benefits, as compared to trees and shrubs. This observation corresponds to previous studies conducted in the other areas of Kohistan [79, 89].

### Part(s) used, modes of preparation and application

The inhabitants of Kohistan use different parts of the reported WMPs to treat various ailments (Fig. 4B). Among these, aerial parts of 36 herbaceous plant species are used in traditional therapies, followed by leaves of 28 species, fruits and roots of 18 species each, branches of 8 species, and stem of 5 species, whereas bark, seeds, rind, flowers, and resin of less than 5 WMPs are used in the primary health care system of Kohistan. Among these, leaves are the most utilized plant parts in traditional remedies for treating various diseases.

The use preference of leaves in traditional therapies could be attributed to their ease of collection compared to other parts of WMPs. Moreover, numerous studies have highlighted that leaves contain a rich concentration of secondary metabolites like polyphenolics, alkaloids, carotenoids, and vitamins, which have significant antimicrobial, antioxidant, anti-inflammatory, and other bioactive properties [89, 90]. Furthermore, the use of leaves instead of roots, flowers, seeds, and fruits in traditional medicines represents a more sustainable conservation approach that minimizes the risk of depleting valuable medicinal plant species [35].

**Fig. 5:**
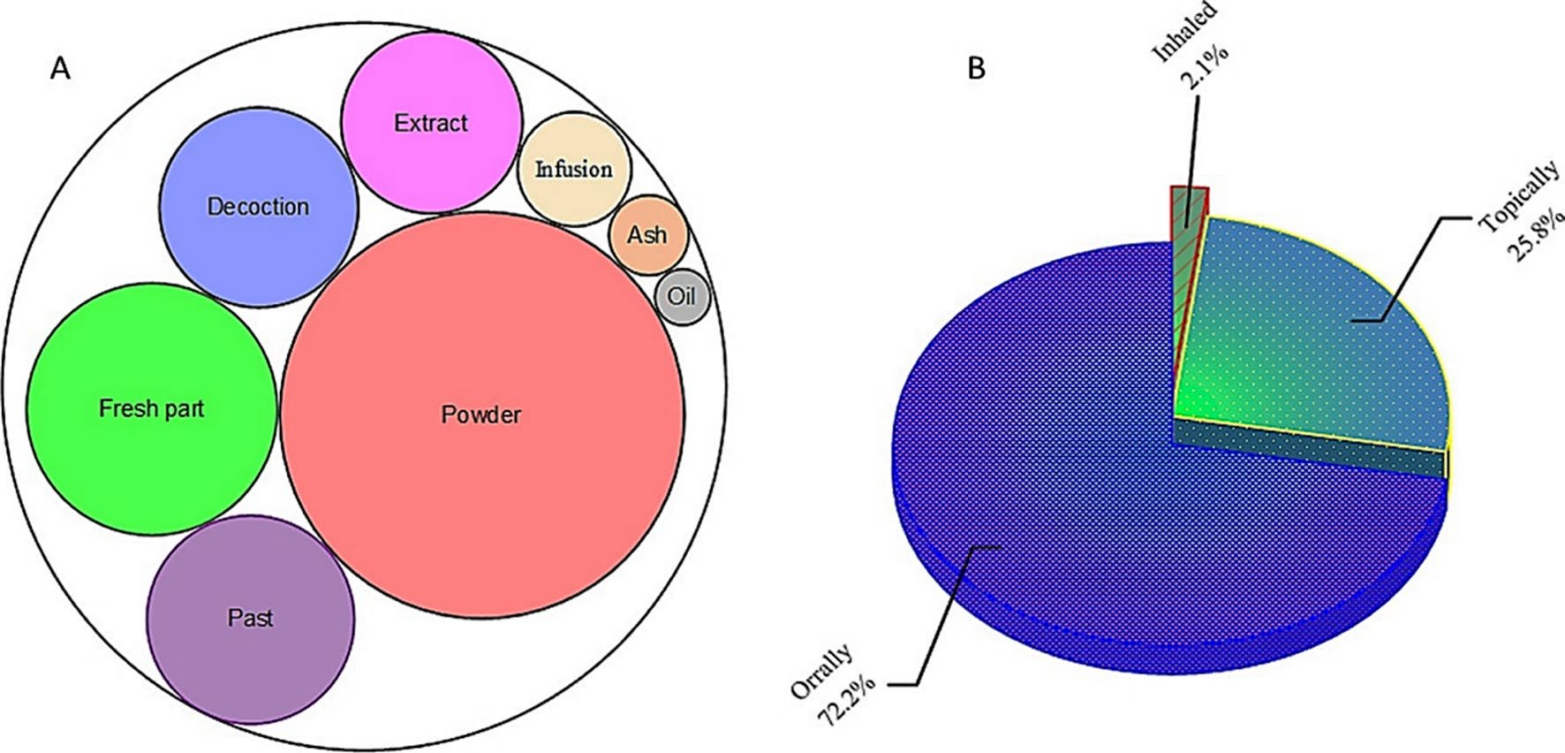
**A** Preparation and **B** administration methods of traditional health care recipes

The array of methods employed in the preparation of herbal remedies includes everything from sun drying and grinding with mortars and pestles, to fermentation, distillation, and maceration, each having its unique advantages. As illustrated in Fig. 5A, local inhabitants of the study area utilize a total of eight different methods for preparing herbal remedies. Most of the reported WMPs (49 species) are shade dried and grinded using a mortar and pestle to make a fine powder that can be taken orally or applied topically to treat various diseases. According to the local respondents, the powdered crude drugs can be stored for extended periods of time in cotton sacks or pots made from mud or silver. Additionally, fresh plant parts (consisting of 19 species), pastes (13 species), decoctions (12 species), and extracts (10 species) are commonly employed modes of preparation in traditional recipes for the treatment of various ailments. These traditional recipes have their own unique preparations and applications. For instance, fresh leaf decoctions are commonly used as cleansing agents, while pastes made from powdered roots are often applied topically to treat skin infections.

Among these recipes, approximately 72% (Fig. 5B) are taken orally in the form of decoctions, infusions, or extracts, while 25.8% are applied topically as pastes, powders, or oils, particularly for addressing skin infections. Water, milk, diluted curd (lassi), ghee, and butter are frequently used as mediums for consuming the powdered or fresh parts of wild medicinal plants. It is worth noting that similar methods of preparation and administration have been reported in previous studies conducted in this region, and neighboring areas [31, 32, 34, 57, 60, 67, 77, 91–94].

In accordance with the guidelines outlined by the international classification of primary care ICPC-2 [95], the recorded ailments among the inhabitants of Kohistan were classified into twelve distinct categories (Table 3). These include general and unspecified diseases (OTHA-A), digestive disorders (GAS-D), cardiovascular problems (CAR-K), conditions related to blood, blood-forming organs, and the immune system (Blood-B), musculoskeletal disorders (SKE-L), neurological disorders (NER-N), respiratory infections (RES-R), skin diseases (DER-S), urological disorders (URO-U), cancer (CAN-C), pregnancies, childbirth, family planning (PRE-W). For diseases that did not neatly fit into any specific category, minor modifications were made. Examples of such cases include fever, allergies, back pain, typhoid, and spiritual uses—these were categorized as general and unspecified.

**Table 3:** Diseases category, informant consent factor, and fidelity level of the reported WMPs

S. #	ICPC category	Ailments	Nbt	Nur	ICF	Botanical taxa	Disease(s) treated	FC	UM	FL
1	OTHA-A: General and unspecified	Fever, allergy, back pain, typhoid, and spiritual uses	13	398	0.97	*Ajuga integrifolia*	Fever	14	11	78.57
2	GAS-D: Digestive problems	Constipation, toothache, intestinal worms, diarrhea, jaundice, indigestion, stomachic pain, tooth gum, liver ailment, appetizer, abdominal warm, hepatitis, ulcer, and obesity	40	114	0.65	*Mentha longifolia*	Diarrhea and indigestion	14	13	92.86
3	CAR-K: Cardiovascular diseases	Blood pressure, and blood purifier	2	73	0.99	*Ziziphus jujuba*	Blood purifier	13	10	76.92
4	Blood-B: Blood, blood-forming organs, and immune mechanism	blood clots and injuries, to stop bleeding	4	117	0.97	*Plocama brevifolia*	Bleeding cuts or wounds	15	7	46.67
5	SKE-L: Musculoskeletal disorders	Joint swelling, and strengthening bones	3	122	0.98	*Datisca cannabina*	Joint swelling	14	8	57.14
6	NER-N: Neurological problems	Paralysis	1	26	1.00	*Indigofera tinctoria*	Paralysis	12	5	41.67
7	RES-R: Respiratory disorders	Flu, coughing, corona viral infection, chest pain, asthma, colds, bronchitis, throat infection, influenza, and chest pain	17	479	0.97	*Lepidium sativum*	Asthma	14	9	64.29
8	DER-S: Skin diseases	Wound healing, eczema, skin rash, skin patches, skin irritation, dandruff, warts, face stain, skin dryness, skin infection, and to remove mol	19	549	0.97	*Myrtus communis*	Eczema	18	17	94.44
9	URO-U: Urological disorders	Urinary disorder, bladder infection, kidney problem, urinary tract infection and urinary disorders	5	119	0.97	*Clematis grata*	Urinary disorder	12	8	66.67
10	CAN-C: Cancer	Tumor	1	26	1.00	*Pteridium esculentum*	Tumor	12	5	41.67
11	PRE-W: Pregnancy, childbearing, family planning	Labor pain	1	26	1.00	*Verbascum thapsus*	Labor pain, Respiratory disorders	12	7	58.33
12	OTH: Others	Anti-rodents, mosquito & mouse repellent, anti-insecticidal and fungicidal, livestock diseases, poultry ailments, and antimicrobial	19	431	0.96	*Ambrosia artemisiifolia*	Anti-rodents, mosquito repellent	14	9	64.29

*Nbt* number of botanical taxa used, *NUR* number of use reports, *PS* prefer species, *FC* frequency of citation, *UM* use mention, *FL* fidelity level

### Quantitative analysis of the reported WMPs

In total 281, respondents of five linguistic groups reported medicinal uses of 96 wild plant species, and total use reports were 3298 (Table 2). Among the reported botanical taxa, *Myrtus communis* had the highest RFC (0.34). This medicinal plant species is evergreen and local inhabitants of Kohistan use its leaves to treat eczema. In addition, *M. communis* is also used as tea, and flavoring agent in traditional cuisines of Kohistan (Amin et al. [1]) and has been reported as a medicinal plant in the neighboring regions [96, 97]. Beside this, *Sambucus wightiana, Punica granatum, Quercus semecarpifolia, Astragalus anisacanthus, Persicaria capitata, Polygonum aviculare,* and *Polypodium sibiricum* had maximum RFC.

The Informant Consent Factor (ICF) specifies consistency of understanding among respondents regarding ethnomedicinal application of specific plant species to cure diseases. The ICF values range from 0 to 1, and a disease category with highest ICF value exhibits maximum consensus of the respondents [98]. The ICF values for various disease categories as mentioned in Table 3 were calculated based on ethnomedicinal information provided by the respondents of different linguistic groups of Kohistan. The ICF values of reported disease categories ranged from 0.65 to 1. The highest number of WMPs were reported against GAS-D disease category, followed by DER-S, OTH, RES-R and OTHA-A (ranged from 13 to 40 botanical species). On the other hand, less than 10 species were documented for the remaining disease categories. Likewise, based on use reports major disease categories were in following order: DER-S ≥ RES-R ≥ OTH ≥ OTHA-A ≥ SKE-L ≥ URO-U ≥ Blood-B ≥ GAS-D ≥ CAR-K ≥ NER-N ≥ CAN-C ≥ PRE-W (Table 3). These findings provide valuable insights into the ethnomedicinal practices within different linguistic groups of Kohistan and highlight which diseases have received more attention in terms of herbal remedies. Overall, findings of this study demonstrate the consistency and agreement among respondents regarding the traditional therapeutic uses of specific plant species for various diseases. The results shed light on potential sources for further exploration and development in ethnobotanical research.

Although the NER-N, CAN-C, and PRE-W disease categories showed the highest ICF values (1 for each category), there was a noticeable lack of WMPs usage (1 species for each disease category) and minimal use reports (26 use reports for each disease category). This could be due to the rarity of neurological disorders (NER-N) and cancer (CAN-C) in the study area or a lack of awareness among the local population about these diseases. In the Kohistan region, neurological disorders such as epilepsy, stroke, and trauma are relatively uncommon. Similarly, cancer is also rare, which could be attributed to under-diagnosis or under-reporting. However, it is important to note that certain regions have a high incidence rate of neurological disorders like epilepsy, while globally there is an increasing trend in cancer cases. Even in developed countries, neurological disorders and cancers contribute significantly to morbidity rates. The difference in reported usage might be attributed to limited accessibility or availability of WMPs, cultural taboos, or a lack of recognition by local healers. Nonetheless, it is worth mentioning that despite being the less common ailments in this region, both CAN-C and PRE-W disease categories still showed high ICF values which indicate the perceived utility of WMPs in this region.

While digestive problems (GAS-D), skin diseases (DER-S), and respiratory system disorders (RES-R) have lower ICF values, they still hold significance in the study area. This is evident from the utilization of a high number of botanical taxa for the treatment of these diseases. For instance, 40 WMPs are used to treat digestive problems, 19 species to cure skin diseases, and 17 species for treating respiratory system disorders (Table 2). The largest number of botanical taxa against the above-mentioned diseases revealed prevalence of such heath disorders in the study area, and acceptability of WMPs among different linguistic groups of Kohistan. Our findings align with previous studies [99–102], reporting digestive disorders and skin infections as common health issues in high mountain regions. Similarly, the harsh climatic conditions, high altitude, exposure to UV radiation, and unhygienic practices at both individual and community levels may contribute to the prevalence of these diseases in Kohistan.

Female health issues, particularly during pregnancy and childbirth (PRE-W), are most prevalent in Kohistan. However, due to cultural barriers, collecting information on these diseases is challenging. In certain societies like Kohistani communities, there is a cultural taboo associated with discussing women’s health problems, making it even more difficult to gather data. These practices hinder our understanding of the true extent of female health issues in this region. Additionally, family planning issues are not common as having more children is preferred in Kohistan. Therefore, it is essential to address these challenges and find ways to gather accurate information on female health issues in this region. And that can be only possible by the active participation of female researchers from Kohistan and its allied areas. By doing so, we can develop effective interventions and provide appropriate healthcare services to improve the well-being of women in the Kohistan region.

**Fig. 6:**
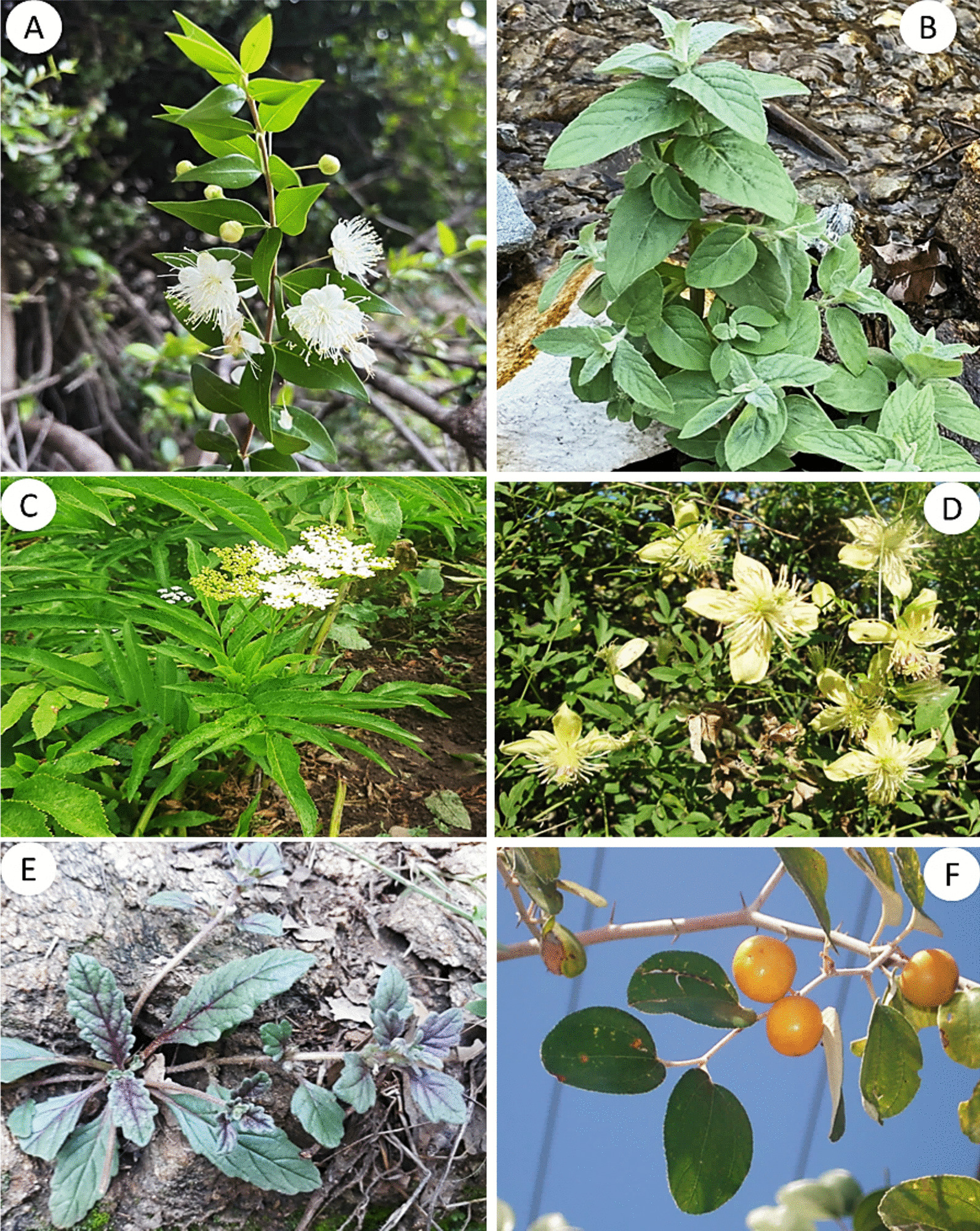
Some highly utilized medicinal plant species **A** *Myrtus communis*, **B** *Mentha longifolia*, **C** *Sambucus wightiana*, **D** *Clematis grata*, **E** *Ajuga integrifolia* **F** *Ziziphus jujuba*

According to Chen et al. [98], the fidelity level (FL) is an important measure for determining the effectiveness of a medicinal plant species in treating specific diseases compared to other plants used for the same purpose. High fidelity levels indicate that a particular plant species is consistently used by many individuals to treat a particular disease [102]. In our study, we found that 12 WMPs had fidelity levels ranging from 41.67% to 94.44%, demonstrating their significant medical applications within different linguistic groups of Kohistan (Table 3). The highest fidelity level of 94.44% was observed for *Myrtus communis* in treating skin diseases, specifically eczema, followed by *Mentha longifolia* with a fidelity level of 92.86% (Fig. 6), commonly used for digestive disorders such as diarrhea and indigestion (GAS-D). Interestingly, this finding aligns with Ahmad et al. [103] report on local communities in Madyan valley, Swat Pakistan, where similar species was utilized against gastrointestinal disorders. While it appears that *M. communis* has not been previously documented for its effectiveness in skin diseases. However, Haq [69] reported that inhabitants of Allai valley in the western Himalayan region of Pakistan use the same species to alleviate bronchial congestion. Likewise, *Ajuga integrifolia, Ziziphus jujuba, Clematis grata, Lepidium sativum, Artemisia brevifolia, Verbascum thapsus,* and *Datisca cannabina* have shown fidelity levels of ≥ 60% against various diseases (Table 3). The inhabitants of Kohistan utilize the leaves of *A. integrifolia* to alleviate fever, skin infections, and purify blood. Conversely, Ozturk et al. [24] stated that the same plant species is used to address hypertension in different regions of Turkey, Pakistan, and Malaysia. Similarly, Muhammad et al. [104] have reported a fidelity level of 100% for *Z. jujuba* from Malakand division KP, Pakistan in relation to lactation support and the treatment of skin disorders, gastrointestinal issues, urological conditions respiratory ailments diabetes and insomnia.

Conversely, the local population in Kohistan employ powdered seeds from *Z. jujuba* to manage blood pressure levels and promote blood purification. Leaf powder from *Clematis grata* is orally administered for the treatment of urological disorders, but Rehman et al. [32] have highlighted that same species is used among tribal communities in the Buner region of Pakistan for managing skin infections. The efficacy of decoctions made from *L. sativum* leaves against asthma aligns with reports by Alamgeer et al. [50]. Inhabitants of Kohistan frequently utilize leaf powder derived from *A. artemisiifolia* as an anti-rodent and mosquito repellent, and its fidelity level is 64.29%. It should be noted that this utilization contradicted to the findings of Zhao et al. [25] who classify *A. artemisiifolia* as an invasive weed that poses harm to crops in Europe and Asia. Moreover, this species is also known to produce copious amounts of allergenic pollen grains that can adversely affect human health [105]. The fidelity level of *V. thapsus* in alleviating labor pain and respiratory disorders was found to be 58.33%, while *D. cannabina* demonstrated has FL 57.14% in reducing joint swelling. It is worth noting that Khan et al. [3] have documented the use of *V. thapsus* leaves for treating asthma and skin infections among the residents of Kashmir Himalayas, Pakistan. However, the application of this plant species against labor pain remains relatively unexplored in existing local and regional literature. Based on the high FL, we recommend further pharmacological investigations on these WMPs to explore their potential benefits and mechanisms of action.

### Cross-culture analysis on the use of the botanical taxa

**Fig. 7:**
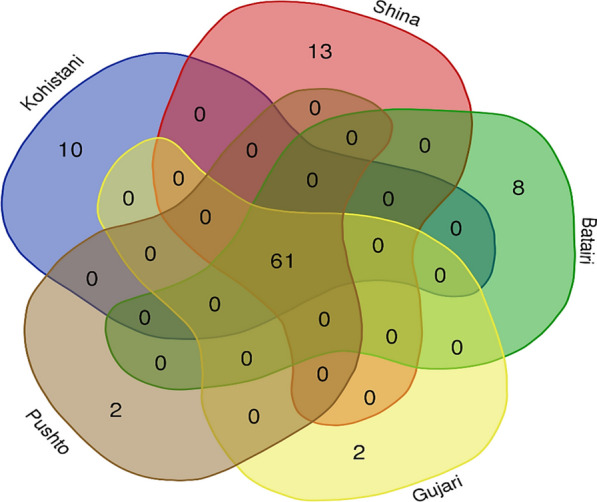
Medicinal plant taxa reported by all linguistic groups

The cross-cultural analysis conducted among the five linguistic groups in Kohistan has unveiled a notable level of heterogeneity on the medical ethnobotany of the studied groups. Considering the number of plant taxa, the quoted botanical taxa among different linguistic groups are illustrated in Fig. 7. A total of 61 plant taxa (63%) were found to be commonly shared across all groups for the treatment of various health issues. However, a noteworthy exception was found in the Shina community, residing in the Himalayan sites of Kohistan, which reported the highest number of unique medicinal plant taxa. The unique taxa reported by the different groups are as follows: Shina: *Aconitum chasmanthum, Juniperus excelsa, Leontopodium himalayanum, Oxyria digyna, Pedicularis oederi, Primula elliptica, P. macrophylla, Pteridium aquilinum, Rheum emodi, Rhodiola integrifolia, Ricinus communis, Rumex nepalensis,* and *Taraxacum campylodes* (refer to Table 2 for detailed information). Bateri: *Buxus wallichiana, Daphne mucronate, Dysphania botrys, Hypericum oblongifolium, Silene conoidea, Solanum virginianum, Sonchus arvensis,* and *Zanthoxylum armatum.* Gujars: *Caltha palustris* and *Cirsium arvense*. Pushton: *Artemisia gmelinii* and *A. stechmanniana*. Kohistani: *Clematis grata, Clinopodium vulgare, Persicaria capitata, Polygonatum multiflorum, Polygonum aviculare, Polypodium sibiricum, Rubus fruticosus, Rumex abyssinicus, Solanum miniatum,* and *Bistorta amplexicaulis*.

**Fig. 8:**
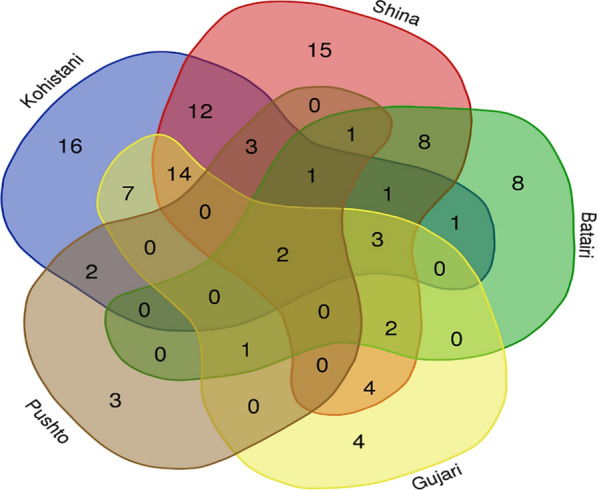
Comparison of medicinal uses mentioned by different linguistic groups

The complex web of medicinal uses of the commonly used plants among the various groups is shown in Fig. 8. Venn diagram elucidates the intricate tapestry of medicinal plant utilization within these linguistic communities. Out of the total 109 documented medicinal uses, approximately 1.83% of the quoted uses were found to be shared across all linguistic groups. We have observed that 12.84% of the uses do overlap among the plant uses of Kohistani, Shina, and Gujjari. It is also important that close affinities (36 uses commonly along 33.02%) on the uses lie between Shina and Kohistani and this may be due their cultural and social dominancy and their social interactions might lead them to share and retain knowledge.

Most of the communal land is owned by these two groups in Kohistan and therefore is evident that these have been the original inhabitants of the study area for generations. The Kohistani and Shinaki people have shared sufficient idiosyncratic uses of plant species, while Bateri also emerged along with them. The Bateri linguistic group who claim to be autochthonous to the study area have some interactions with Kohistani, and we have observed the names of some plants who shared them with Shina although the fact is that their language is branch of Kohistani languages. Despite claiming indigenous roots in Kohistan, the Bateri population has diminish gradually in size over time and now only concentrated in the Bateri village, nestled within the Himalayan expanse of Kohistan-an area. Although Bateri is a linguistic minority in Kohistan, the distinctive wealth of traditional knowledge on idiosyncratic medicinal uses of wild plant species among them can be attributed to their ancestral knowledge, strong association with traditional health care system, historical marginalization, and to some extent due to intermarriage with Kohistani and Shinaki communities.

Within the broader context of Kohistan, Shina speakers (Shinaki) and Kohistani constitute the predominant linguistic groups, collectively spanning almost 80% of the region. The Shinaki groups assert dominance in the Himalayan regions, while the Kohistani communities prevail along the Hindu Kush Mountain range, flanking the right and left banks of the Indus River, respectively. Despite the abundant diversity of medicinal plants in both mountainous terrains, the Kohistani and Shina groups reported relatively fewer medicinal uses, documenting 27 and 26, respectively. As reported earlier [106, 107], discernible impact of modernization, encapsulating factors such as enhanced education, migration, and urbanization, is evident within Kohistan, and Shina linguistic groups is leading to a discernible decline in traditional knowledge. Adding a layer of complexity, the migratory patterns of the Shinaki people of Jalkot and allied areas offer insights into their traditional knowledge. During the summer, these communities typically migrate toward subalpine and alpine pastures where the above-mentioned botanical taxa are commonly found. As a result, the Shinaki people possess an intimate understanding of the medical applications of these unique plant species prevalent in the alpine and subalpine regions. The Pushton and Gujjari communities, often perceived as non-native in Kohistan, are confined to specific geographical areas. The Gujjari people, primarily nomadic, exhibit a preference for alpine and subalpine valleys and pastures. In contrast, a limited population of Pushto speakers resides in the lower regions of Kohistan along the River Indus. This limited dissemination of traditional knowledge is attributed to the tendency of Gujjari community to preserve such knowledge within the confines of their own family circles. The exploration of plant resource utilization among diverse ethnolinguistic groups unveils a fascinating tapestry of both homogeneity and heterogeneity [1, 108–110].

### Novelty in reported WMPs

**Fig. 9:**
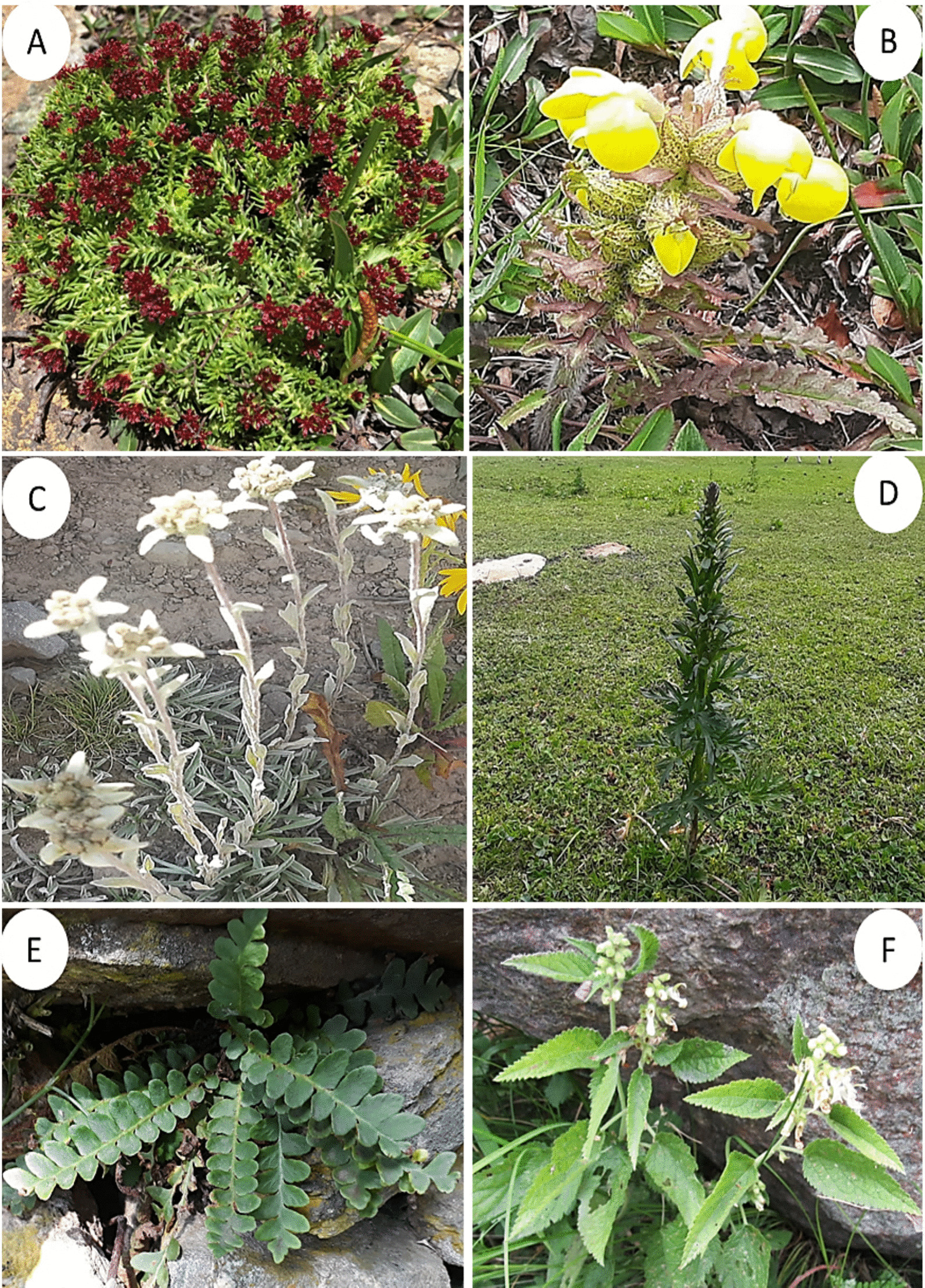
Some newly reported medicinal plants of Kohistan. **A** *Rhodiola integrifolia*, **B** *Pedicularis oederi*, **C** *Leontopodium himalayanum*, **D** *Aconitum chasmanthum*, **E** *Pteridium esculentum*, **F** *Teucrium royleanum*

Comparative assessment of ethnomedicinal uses reported by different linguistic groups of Kohistan (as shown in Table 2) with previously reported literature revealed that out of ninety-six documented WMPs about 90% have already been reported from different areas of Pakistan and neighboring regions. However, to best of our knowledge, 10 botanical taxa, namely *Leontopodium himalayanum*, *Pedicularis oederi*, *Plocama brevifolia*, *Polypodium sibiricum*, *Pteridium esculentum*, *Sambucus wightiana*, *Solanum cinereum*, *Teucrium royleanum*, *Rhodiola integrifolia*, and *Aconitum chasmanthum*, have been reported for the first time (Fig. 9). In addition, although *Artemisia stechmanniana, Cirsium verutum, Rubus niveus, Rumex abyssinicus*, and *Silene conoidea*, are used in traditional health care systems of China, Nepal, Bangladesh, Ethiopia, and India [27, 36, 66, 68, 71], they never have been reported as medicinal plant species in Pakistan. Likewise, there were significant variations in plant part(s) used, mode of preparation, application, and types of diseases treated of the commonly reported medicinal plant species. For instance, the aerial parts of *Primula elliptica* and *P. macrophylla* are used in snuff by the inhabitants of Kohistan, but roots and flowers of the same species are used to heal wounds and against jaundice in Swat, Pakistan [61]. Likewise, inhabitants of Kohistan use seeds of *Ziziphus jujuba* as blood purifier; however, fruits and roots of same species were reported to treat diabetes and obesity [53, 79].

### Threats to ethnomedicinal knowledge of Kohistan

Ethnomedicinal knowledge among various linguistic groups reflects their beliefs, cultural practices, and bioresource management experience [111]. This knowledge, especially among minor groups in mountain regions like northern parts of Pakistan, is at risk of erosion due to challenges like globalization and urbanization [13, 99]. Our research found that the Bateri, a minor group, has significant botanical knowledge for public health, but it is fading due to younger generations’ disinterest and influence from dominant groups like Shina and Kohistani. This loss threatens traditional ethnomedicinal knowledge from this region.

In Kohistan, mega projects like the Dassu and Diamar Bhasha dams are changing lifestyles, leading to cultural erosion and loss of indigenous knowledge. It has also been seen that people migrate to other areas and the economic situation among many households is in transition. Because of this Kohistani people are losing connection to their local natural resources that in turn impact the core body of knowledge and its intergenerational transmission. Young people relocating for better job opportunities further disconnect them from their traditional roots. One of the most important things is that Shina and Kohistani who are the dominant groups in the study area are somehow going through the economic transition as the developmental infrastructures are affecting the localities of the two groups. Therefore, their plant knowledge transmission is more threatened than other groups. Overall, the practical use of local medicinal plants is sufficiently practiced as compared to other parts of the surrounding areas [21, 60, 88, 109, 112].

The absence of written documentation and inclusion in educational curricula exacerbates this loss. We can also use this to make people aware of the importance of natural resources, brochures should be periodically circulated. These brochures should be properly designed, and the relevant ethnobotanical knowledge should be the main material included in that. These brochures should be part of workshops that address the conservation of local flora in schools and colleges in the study area. Thus, preserving this knowledge requires a holistic approach involving government and educational authorities, focusing on conservation, natural resource management, and incorporating traditional knowledge into local education [99]. Moreover, engaging local communities and religious schools in Kohistan is crucial for the success of these conservation efforts, as these communities highly regard instructions from religious leaders. Cultural activities and educational programs can also aid in preserving this knowledge.

## Conclusions

The present study was focused on the documentation of ethnomedicinal knowledge on wild plant species in Kohistan and its cross-linguistic comparison among different communities. Comparative assessment revealed some novel botanical taxa and medicinal uses which are valuable additions into the existing stock of ethnomedicinal knowledge and may provide ethnopharmacological basis for novel drug discovery and therapeutic opportunities for preexisting and emerging diseases. In the present study, a compelling observation of significant homogeneity emerged in the reported botanical taxa among different linguistic groups, and ≥ 63% of the plant species, possessing diverse therapeutic properties, were commonly employed across all groups for the treatment of various health disorders. Surprisingly, the Bateri linguistic group (a minority) emerges as a notable outlier, detailing the highest number of medicinal uses of the reported botanical taxa, and the distinctive wealth of traditional knowledge on idiosyncratic medicinal uses of wild plant species among them can be attributed to their ancestral knowledge, strong association with traditional health care system, historical marginalization, and to some extent due to intermarriage with Kohistani and Shinaki communities. Our findings revealed that in the era of modernization, Kohistan still holds a unique biocultural diversity thus proving our hypothesis on rich floristic and cultural diversity of Kohistan and significant ethnomedicinal knowledge of its inhabitants. However, changing live style, shifting socio-economic circumstances, cross-cultural interactions, migration due to remoteness, dominance of major linguistic groups, oral transmission, and lack of proper documented ethnomedicinal knowledge, and ignorance of government and local authorities are multifaceted risks to traditional knowledge and biocultural heritage of Kohistan. In this context, public awareness and education of young generation by the active involvement of education systems “formal and religious” may contribute significantly to the protection, and conservation of biocultural diversity, and ethnolinguistic knowledge in Kohistan. Women in the mountain regions are main custodians of ethnomedicinal knowledge and hence must be given priority in future studies in Kohistan region. Furthermore, phytochemical composition and pharmacological studies should consider wild medicinal plant species of Kohistan having highest RFC, ICF, and FL values.

## Data Availability

All data are available in this paper.

## Abbreviations

EMK: Ethnomedicinal knowledge
MPs: Medicinal plant species
FDA: Food and drug administration
KP: Khyber Pakhtunkhwa
CPEC: China–Pakistan economic corridor
RFC: Relative frequency citation
ICF: Informant consent factor
FL: Fidelity level
WMPs: Wild medicinal plant species

## References

[1] Amin M, Aziz MA, Pieroni A, Nazir A, Al-Ghamdi AA, Kangal A, Abbasi AM (2023). Edible wild plant species used by different linguistic groups of Kohistan Upper Khyber Pakhtunkhwa (KP). Pak J Ethnobiol Ethnomed.

[2] Khan A, Ahmed M, Siddiqi MF, Shah M, Calixto ES, Khan A, Azeem M (2020). Vegetation-environment relationship in conifer dominating forests of the mountainous range of Indus Kohistan in northern Pakistan. J Mt Sci.

[3] Khan A, Ahmed M, Siddiqui MF, Shah M, Hazrat A (2021). Quantitative description, present status and future trend of conifer forests growing in the Indus Kohistan region of Khyber Pakhtunkhwa. Pak Pak J Bot.

[4] Ismail I, Sohail M, Gilani H, Ali A, Hussain K, Hussain K, Kotru R (2018). Forest inventory and analysis in Gilgit-Baltistan: a contribution towards developing a forest inventory for all Pakistan. Int J Clim Change Strateg Manag.

[5] Khan A, Ahmed M, Siddiqui MF, Iqbal J, Wahab M (2016). Phytosociological analysis of Pine Forest at Indus Kohistan, KPK. Pak Pak J Bot.

[6] Aati H, El-Gamal A, Shaheen H, Kayser O (2019). Traditional use of ethnomedicinal native plants in the Kingdom of Saudi Arabia. J Ethnobiol Ethnomed.

[7] Rensch CR (1992). Patterns of language use among the Kohistanis of the Swat valley. Sociolinguistic Surv Northern Pak.

[8] Zoller CP (2005). A grammar and dictionary of Indus Kohistani.

[9] Liljegren H (2019). Gender typology and gender (in) stability in Hindu Kush Indo-Aryan languages. Gramm Gender Linguist Complex.

[10] Frembgen JW (1999). Indus kohistan an historical and ethnographie outline. Cent Asiat J.

[11] Hallberg DG. The languages of Indus Kohistan. Languages of Kohistan. In SSNP-1 1992;83–141.

[12] Schmidt RL, Zarin MM (1981). The phonology and tonal system of Páalus/kohis' tyó~:/Shina. Münch Stud Sprachwiss.

[13] Issa M, Khan HK, Hussain MS, Ali MA (2023). Language erosion: an overview of declining status of indigenous languages of Gilgit-Baltistan. Pak Int J Multicult Educ.

[14] Ali SS (2020). Linguistic landscape and the public space: a case study of GilgitBaltistan. Kashmir J Lang Res.

[15] Schmidt RL (2004). A grammatical comparison of Shina dialects. Trends Linguist Stud Monogr.

[16] Grierson GA. The ormuri or bargista language. Mem Asiat Soc Bengal 1918;7(1).

[17] Oleary CF (1992). Sociolinguistic survey of northern Pakistan (V1 to V5).

[18] ISE. International society of ethnobiology. Code of ethics. 2016. https://www.ethnobiology.net/what-we-do/core-programs/ise-ethics-program/code-of-ethics/. Accessed 1 Oct 2022

[19] Delpero A, Volpato G (2022). Integrated pond aquaculture and regional identity: ethnobiology of the golden humped tench of Poirino highlands, Northwest Italy. J Ethnobiol Ethnomed.

[20] Aziz MA, Ullah Z, Al-Fatimi M, De Chiara M, Sõukand R, Pieroni A (2021). On the trail of an ancient Middle Eastern ethnobotany: traditional wild food plants gathered by Ormuri speakers in Kaniguram. NW Pak Biol.

[21] Aziz M, Abbasi AM, Ullah Z, Pieroni A (2020). Shared but threatened: The heritage of wild food plant gathering among different linguistic and religious groups in the Ishkoman and Yasin Valleys, North Pakistan. Foods.

[22] Aziz MA, Ullah Z, Adnan M, Sõukand R, Pieroni A (2022). Plant use adaptation in Pamir: Sarikoli foraging in the wakhan area. Northern Pak Biol.

[23] Ali, S. I. and Qaiser, M. eds. Flora of Pakistan. Karachi: University of Karachi; 1993–2009.

[24] Ozturk M, Altay V, Latiff A, Shareef S, Shaheen F, Iqbal CM (2018). Potential medicinal plants used in the hypertension in Turkey, Pakistan, and Malaysia. Plant Hum Health Vol Ethnobot Physiol.

[25] Zhao W, Liu T, Sun M, Wang H, Liu X, Su P (2022). Rapid monitoring of *Ambrosia artemisiifolia* in semi-arid regions based on ecological convergence and phylogenetic relationships. Front Ecol Evol.

[26] Khan SW, Khatoon S (2004). Ethnobotanical studies in Haramosh and Bugrote valleys (Gilgit). Int J Biotechnol.

[27] Kim WH, Jang HJ, Kim J, Jeong CH, Enebish G, Kim SY, Min H (2021). Anti-inflammatory effects of *Artemisia stechmanniana* Besser extract on LPS-stimulated macrophages. Food Hydrocoll.

[28] Abidin SZU, Munem A, Khan R, Batiha GES, Amhad M, Zafar M, Bhatti MZ (2021). Ethnoveterinary botanical survey of medicinal plants used in Pashto, Punjabi and Saraiki communities of Southwest Pakistan. Vet Med Sci.

[29] Ahmad M, Zafar M, Shahzadi N, Yaseen G, Murphey TM, Sultana S (2018). Ethnobotanical importance of medicinal plants traded in Herbal markets of Rawalpindi-Pakistan. J Herbal Med.

[30] Rashid N, Gbedomon RC, Ahmad M, Salako VK, Zafar M, Malik K (2018). Traditional knowledge on herbal drinks among indigenous communities in Azad Jammu and Kashmir. Pak J Ethnobiol Ethnomed.

[31] Sulaiman, Shah S, Khan S, Bussmann RW, Ali M, Hussain D, Hussain W. Quantitative ethnobotanical study of Indigenous knowledge on medicinal plants used by the tribal communities of Gokand Valley, District Buner, Khyber Pakhtunkhwa, Pakistan. Plants 2020;9(8):1001. 10.3390/plants9081001 PMC7463859 32781736

[32] Rehman S, Iqbal Z, Qureshi R, Ur Rahman I, Khan MA, Elshaer M, Abu Bakr Elsaid NM (2022). Ethnogynaecological knowledge of traditional medicinal plants used by the indigenous communities of north Waziristan, Pakistan. Evid-Based Complement Altern Med.

[33] Ayoub M, Saeed M, Saeed S, Saeed A, Ahmad M. Ethnobotanical survey and utilization of medicinal and food plants of Panjgur Balochistan, Pakistan. Asian J Ethnobiol 6(1).

[34] Haq F, Ahmad H, Alam M (2011). Traditional uses of medicinal plants of Nandiar Khuwarr catchment (District Battagram). Pak J Med Plants Res.

[35] Umair M, Altaf M, Abbasi AM (2017). An ethnobotanical survey of indigenous medicinal plants in Hafizabad district. Punjab-Pak PloS One.

[36] Adhikari M, Thapa R, Kunwar RM, Devkota HP, Poudel P (2019). Ethnomedicinal uses of plant resources in the Machhapuchchhre rural municipality of Kaski District, Nepal. Medicines.

[37] Ajaib M, Ishtiaq M, Bhatti KH, Hussain I, Maqbool M, Hussain T, Bashir R (2021). Inventorization of traditional ethnobotanical uses of wild plants of Dawarian and Ratti Gali areas of District Neelum, Azad Jammu and Kashmir Pakistan. PLoS ONE.

[38] Afzal S, Afzal N, Awan MR, Khan TS, Gilani A, Khanum R, Tariq S (2009). Ethno-botanical studies from Northern Pakistan. J Ayub Med Coll Abbottabad.

[39] Ahmed E, Arshad M, Saboor A, Qureshi R, Mustafa G, Sadiq S, Chaudhari SK (2013). Ethnobotanical appraisal and medicinal use of plants in Patriata, New Murree, evidence from Pakistan. J Ethnobiol Ethnomed.

[40] Sharif A, Asif H, Younis W, Riaz H, Bukhari IA, Assiri AM (2018). Indigenous medicinal plants of Pakistan used to treat skin diseases: a review. Chin Med.

[41] Younis W, Asif H, Sharif A, Riaz H, Bukhari IA, Assiri AM (2018). Traditional medicinal plants used for respiratory disorders in Pakistan: a review of the ethno-medicinal and pharmacological evidence. Chin Med.

[42] Iqbal MS, Ahmad KS, Ali MA, Akbar M, Mehmood A, Nawaz F, Bussmann RW (2021). An ethnobotanical study of wetland flora of Head Maralla Punjab Pakistan. PLoS ONE.

[43] Arshad M, Ahmad M (2005). Ethnobotanical study of Galliyat for botanical demography and bioecological diversification. Ethnobotan Leafl.

[44] Hussain S, Hamid A, Ahmad KS, Mehmood A, Nawaz F, Ahmed H (2019). Quantitative ethnopharmacological profiling of medicinal shrubs used by indigenous communities of Rawalakot, District Poonch, Azad Jammu and Kashmir, Pakistan. Rev Bras.

[45] Ahsan AAMUH, Chaudhary UHHMA (2018). Traditional medicines of plant origin used for the treatment of inflammatory disorders in Pakistan: a review. J Tradit Chin Med.

[46] Amber R, Adnan M, Tariq A, Mussarat S (2017). A review on antiviral activity of the Himalayan medicinal plants traditionally used to treat bronchitis and related symptoms. J Pharm Pharmacol.

[47] Rahman IU, Ijaz F, Iqbal Z, Afzal A, Ali N, Afzal M, Asif M (2016). A novel survey of the ethno medicinal knowledge of dental problems in Manoor Valley (Northern Himalaya), Pakistan. J Ethnopharmacol.

[48] Nabi S, Al-Kahraman YM, Tahira B, Hajira B, Rasool A, Muhammad A (2017). A review on Juniperus Excelsa: description, distribution and ecology, ethnobotany, and biological activities. Indo Am J Pharm Sci.

[49] Adnan M, Bibi R, Mussarat S, Tariq A, Shinwari ZK (2014). Ethnomedicinal and phytochemical review of Pakistani medicinal plants used as antibacterial agents against *Escherichia coli*. Ann Clin Microbiol Antimicrob.

[50] Alamgeer Uttra AM, Ahsan H, Hasan UH, Chaudhary MA (2018). Traditional medicines of plant origin used for the treatment of inflammatory disorders in Pakistan: a review. J Tradit Chin Med.

[51] Rehman S, Iqbal Z, Qureshi R, Shah GM (2023). Quantitative ethnobotanical study of medicinal plants used by the indigenous communities of Shawal Valley, District North Waziristan, Pakistan. Ethnobot Res Appl.

[52] Dutta A, Sharma YP, Bikarma BS, Bussmann RW (2022). Plant-based veterinary practices in Jammu and Kashmir: a review of the trends, transfer, and conservation of traditional ethnoveterinary knowledge. Ethnobot Res Appl.

[53] Wali R, Khan MF, Mahmood A, Mahmood M, Qureshi R, Ahmad KS, Mashwani ZUR (2022). Ethnomedicinal appraisal of plants used for the treatment of gastrointestinal complaints by tribal communities living in Diamir district, Western Himalayas. Pakistan Plos One.

[54] Batool S, Ahmed MS (2016). *Bistorta amplexicaulis*: a brief insight to its ethnobotany. J Bioresour Manag.

[55] Natarajan B, Paulsen BS, Korneliussen V (2000). An ethnopharmacological study from Kulu District, Himachal Pradesh, India: traditional knowledge compared with modern biological science. Pharm Biol.

[56] Khan SW, Khatoon S (2007). Ethnobotanical studies on useful trees and shrubs of Haramosh and Bugrote valleys in Gilgit northern areas of Pakistan. Pak J Bot.

[57] Jan G, Khan MA, Gul F (2008). Ethnomedicinal plants used against diarrhea and dysentery in Dir Kohistan valley (NWFP). Pak Ethnobot Leafl.

[58] Hamayun M (2007). Traditional uses of some medicinal plants of Swat Valley, Pakistan. Ethnobotanical Leaflets.

[59] Sher H, Elyemeni M, Hussain K, Sher H (2011). Ethnobotanical and economic observations of some plant resources from the northern parts of Pakistan. Ethnobot Res Appl.

[60] Abbasi AM, Khan MA, Shah MH, Shah MM, Pervez A, Ahmad M (2013). Ethnobotanical appraisal and cultural values of medicinally important wild edible vegetables of Lesser Himalayas-Pakistan. J Ethnobiol Ethnomed.

[61] Khan S, Shaheen H, Mehmood A, Nasar S, Khan T (2022). Ethnobotanical and antibacterial study of Primula plants traditionally used in the indigenous communities of Western Himalaya. Pak Saudi J Biol Sci.

[62] Aziz MA, Adnan M, Khan AH, Rehman AU, Jan R, Khan J (2016). Ethno-medicinal survey of important plants practiced by indigenous community at Ladha subdivision, South Waziristan agency, Pakistan. J Ethnobiol Ethnomed.

[63] Hamayun M (2005). Ethnobotany of some useful trees of Hindu-Kush Mountain region: a case study of Swat Kohistan, district Swat. Pak Ethnobot Leafl.

[64] Pandith SA, Dar RA, Lattoo SK, Shah MA, Reshi ZA (2018). *Rheum australe*, an endangered high-value medicinal herb of Northwestern Himalayas: a review of its botany, ethnomedical uses, phytochemistry and pharmacology. Phytochem Rev.

[65] Daniyal M, Tahir IM, Akram M, Zahid R, Zainab R, Riaz Z, Bin L (2019). Pharmacological effects of Rheum emodi: a multiple purpose plant in health and disease. Pak J Med Biol Sci.

[66] Uniyal SK, Singh KN, Jamwal P, Lal B (2006). Traditional use of medicinal plants among the tribal communities of Chhota Bhangal, Western Himalaya. J Ethnobiol Ethnomed.

[67] Jan G, Khan MA, Gul F (2009). Ethnomedicinal plants used against jaundice in Dir Kohistan valleys (NWFP). Pak Ethnobot Leafl.

[68] Woldemariam G, Demissew S, Asfaw Z (2021). An ethnobotanical study of traditional medicinal plants used for human ailments in Yem ethnic group, south Ethiopia. Ethnobot Res Appl.

[69] Haq F (2012). The ethno botanical uses of medicinal plants of Allai Valley, Western Himalaya Pakistan. Int J Plant Res.

[70] Abbas Z, Khan SM, Alam J, Khan SW, Abbasi AM (2017). Medicinal plants used by inhabitants of the Shigar Valley, Baltistan region of Karakorum range-Pakistan. J Ethnobiol Ethnomed.

[71] Chandra S, Rawat DS (2015). Medicinal plants of the family Caryophyllaceae: a review of ethno-medicinal uses and pharmacological properties. Integr Med Res.

[72] Nabi M, Tabassum N, Ganai BA (2022). *Skimmia anquetilia* NP Taylor and airy shaw (rutaceae): A critical appraisal of its ethnobotanical and pharmacological activities. Front Plant Sci.

[73] Abdullah A (2021). A comprehensive appraisal of the wild food plants and food system of tribal cultures in the Hindu Kush Mountain Range; a way forward for balancing human nutrition and food security. Sustainability.

[74] Aziz MA, Khan AH, Adnan M, Ullah H (2018). Traditional uses of medicinal plants used by Indigenous communities for veterinary practices at Bajaur Agency. Pak J Ethnobiol Ethnomed.

[75] Iqbal H, Sher Z (2011). Medicinal plants from salt range Pind Dadan Khan, district Jhelum, Punjab. Pak J Med Plants Res.

[76] Ata S, Farooq F, Javed S (2011). Elemental profile of 24 common medicinal plants of Pakistan and its direct link with traditional uses. J Med Plant Res.

[77] Malik K, Ahmad M, Zafar M, Ullah R, Mahmood HM (2019). An ethnobotanical study of medicinal plants used to treat skin diseases in northern Pakistan. BMC Complement Altern Med.

[78] Iqbal I, Hamayun M (2004). Studies on the traditional uses of plants of Malam Jabba valley, District Swat. Pak Ethnobot Leafl.

[79] Ahmad K, Ahmad M, Huber FK, Weckerle CS (2021). Traditional medicinal knowledge and practices among the tribal communities of Thakht-e-Sulaiman Hills, Pakistan. BMC Complement Med Ther.

[80] Ishtiaq M, Maqbool M, Ajaib M, Ahmed M, Hussain I, Khanam H, Ghani A (2021). Ethnomedicinal and folklore inventory of wild plants used by rural communities of valley Samahni, District Bhimber Azad Jammu and Kashmir. Pak Plos One.

[81] Vitalini S, Iriti M, Puricelli C, Ciuchi D, Segale A, Fico G (2013). Traditional knowledge on medicinal and food plants used in Val San Giacomo (Sondrio, Italy)—An alpine ethnobotanical study. J Ethnopharmacol.

[82] Ong HG, Kim YD (2014). Quantitative ethnobotanical study of the medicinal plants used by the Ati Negrito indigenous group in Guimaras island, Philippines. J Ethnopharmacol.

[83] Friedman J, Yaniv Z, Dafni A, Palewitch D (1986). A preliminary classification of the healing potential of medicinal plants, based on a rational analysis of an ethnopharmacological field survey among Bedouins in the Negev Desert, Israel. J Ethnopharmacol.

[84] Idm'hand E, Msanda F, Cherifi K (2020). Ethnobotanical study and biodiversity of medicinal plants used in the Tarfaya Province, Morocco. Acta Ecol Sin.

[85] Barth F (1956). Indus and swat kohistan-an ethnographic survey.

[86] Gu XJ, Xiao PG (2015). Phytochemical and biological research of Polygoneae medicinal resources. Med Plants.

[87] Noor A, Khatoon S, Ahmed M, Razaq A (2014). Ethnobotanical study on some useful shrubs of Astore valley, Gilgit-Baltistan, Pakistan. Bangladesh J Bot.

[88] Abbas Z, Kousar S, Aziz MA, Pieroni A, Aldosari AA, Bussmann RW, Abbasi AM (2021). Comparative assessment of medicinal plant utilization among Balti and Shina communities in the periphery of Deosai National Park. Pak Biol.

[89] Abbas Q, Khan SW, Khatoon S, Hussain SA, Hassan SN, Hussain A, Hussain I (2014). Floristic biodiversity and traditional uses of medicinal plants of Haramosh valley Central Karakoram National Park of Gilgit district, Gilgit-Baltistan. *Pakistan*. J Biol Environ Sci.

[90] Gang R, Matsabisa M, Okello D, Kang Y (2023). Ethnomedicine and ethnopharmacology of medicinal plants used in the treatment of diabetes mellitus in Uganda. Appl Biol Chem.

[91] Hussain S, Hussain W, Nawaz A, Badshah L, Ali A, Ullah S, Bussmann RW (2022). Quantitative ethnomedicinal study of indigenous knowledge on medicinal plants used by the tribal communities of Central Kurram, Khyber Pakhtunkhwa, Pakistan. Ethnobot Res Appl.

[92] Birjees M, Ahmad M, Zafar M, Nawaz S, Jehanzeb S, Ullah F, Zaman W (2022). Traditional knowledge of wild medicinal plants used by the inhabitants of Garam Chashma valley, district Chitral. Pak Acta Ecol Sin.

[93] Afzal S, Ahmad HI, Jabbar A, Tolba MM, AbouZid S, Irm N, Aslam Z (2021). Use of medicinal plants for respiratory diseases in Bahawalpur, Pakistan. BioMed Res Int.

[94] Jan HA, Jana S, Walia S, Ahmad L, Sisto F, Bussmannd RW, Romman M (2021). Ethnomedicinal study of medicinal plants used to cure dental diseases by the indigenous population of district Buner, Pakistan. Indian J Tradit Knowl.

[95] Verbeke M, Schrans D, Deroose S, De Maeseneer J (2006). The International Classification of Primary Care (ICPC-2): an essential tool in the EPR of the GP. Stud Health Technol Inf.

[96] Dabbaghi MM, Fadaei MS, Soleimani Roudi H, Baradaran Rahimi V, Askari VR (2023). A review of the biological effects of Myrtus communis. Physiol Rep.

[97] Medda S, Mulas M (2021). Fruit quality characters of myrtle (*Myrtus communis* L.) selections: review of a domestication process. Sustainability.

[98] Chen TX, Lia WZ, Hua CG, Chen YL, Zhao TT, Hu JZ, Tang J (2023). Review article ethnobotanical study of miao nationality medicinal plants in Leishan County, Guizhou Province, China. J Herbal Med.

[99] Aziz MA, Hassan M, Ullah A, Ullah Z, Sõukand R, Pieroni A (2023). Keeping their own and integrating the other: medicinal plant use among Ormurs and Pathans in South Waziristan. Pak J Ethnobiol Ethnomed.

[100] Bouafia M, Amamou F, Gherib M, Benaissa M, Azzi R, Nemmiche S (2021). Ethnobotanical and ethnomedicinal analysis of wild medicinal plants traditionally used in Naâma, southwest Algeria. Vegetos.

[101] Amjad MS, Zahoor U, Bussmann RW, Altaf M, Gardazi SMH, Abbasi AM (2020). Ethnobotanical survey of the medicinal flora of Harighal, Azad Jammu & Kashmir, Pakistan. J Ethnobiol Ethnomed.

[102] Tamene S, Addisu D, Debela E (2020). Ethno-medicinal study of plants in Boricha district: Use, preparation and application by traditional healers, Southern Ethiopia. J Med Plants Res.

[103] Ahmad N, Anwar S, Fazal H, Abbasi BH (2013). Medicinal plants used in indigenous therapy by people of Madyan Valley in district Swat, Pakistan. Int J Med Aromat Plants.

[104] Muhammad N, Uddin N, Khan MKU, Mengjun L, Xuan Z, Ali N, Liu Z (2020). Ethnomedicinal and cultural uses of Ziziphus species in flora of Malakand Division KP, Pakistan. Singapore J Sci Res.

[105] Hamaoui-Laguel L, Vautard R, Liu LI, Solmon F, Viovy N, Khvorostyanov D, Epstein MM (2015). Effects of climate change and seed dispersal on airborne ragweed pollen loads in Europe. Nat Clim Chang.

[106] Arjona-García C, Blancas J, Beltrán-Rodríguez L, López Binnqüist C, Colín Bahena H, Moreno-Calles AI, López-Medellín X (2021). How does urbanization affect perceptions and traditional knowledge of medicinal plants?. J Ethnobiol Ethnomed.

[107] Saynes-Vásquez A, Caballero J, Meave AJ, Chiang F (2013). Cultural change, and loss of ethnoecological knowledge among the Isthmus Zapotecs of Mexico. J Ethnobiol Ethnomed.

[108] Yao R, He C, Xiao P (2023). ‘Food and medicine continuum’in the East and West: old tradition and current regulation. Chin Herbal Med.

[109] Haq SM, Hassan M, Bussmann RW, Calixto ES, Rahman IU, Sakhi S, Ali N (2022). A cross-cultural analysis of plant resources among five ethnic groups in the Western Himalayan region of Jammu and Kashmir. Biology.

[110] Majeed M, Bhatti KH, Pieroni A, Sõukand R, Bussmann RW, Khan AM, Amjad MS (2021). Gathered wild food plants among diverse religious groups in Jhelum District, Punjab. Pak Foods.

[111] Pieroni A, Vandebroek I (2009). Traveling cultures and plants: the ethnobiology and ethnopharmacy of human migrations.

[112] Ali A, Badshah L, Hussain F (2018). Ethnobotanical appraisal and conservation status of medicinal plants in Hindukush Range, District Swat, Pakistan. J Herbs Spices Med Plants.

